# Transfer effects of animal-assisted therapy on socioemotional skills in adult inpatient rehabilitation after acquired brain injury: a randomized controlled trial

**DOI:** 10.3389/fresc.2026.1818862

**Published:** 2026-09-03

**Authors:** Elena Pauli, Sophie Dubach, Carmina Haase, Anna Haefeli, Margret Hund-Georgiadis, Jan Hattendorf, Karin Hediger

**Affiliations:** 1 Faculty of Psychology, Division of Clinical Psychology and Psychotherapy, University of Basel, Basel, Switzerland; 2 Clinic for Neurorehabilitation and Paraplegiology, REHAB Basel, Basel, Switzerland; 3 Faculty of Behavioral Sciences and Psychology, Division of Child and Adolescent Psychology, University of Lucerne, Lucerne, Switzerland; 4 University Psychiatric Clinics (UPK), University of Basel, Basel, Switzerland; 5 Biostatistics and Data Management, Swiss Tropical and Public Health Institute, Allschwil, Switzerland; 6 University of Basel, Basel, Switzerland; 7 Faculty of Behavioural and Health Sciences, Open University, Heerlen, Netherlands

**Keywords:** acquired brain injury, animal-assisted therapy, neurorehabilitation, randomized controlled trial, social behavior, socioemotional functioning

## Abstract

**Background:**

People with acquired brain injury (ABI) often experience difficulties in social and emotional functioning, potentially affecting recovery and quality of life. Animal-assisted therapy (AAT) is increasingly used in rehabilitation to support socioemotional functioning.

**Objective:**

This randomized controlled trial investigated whether the effects of AAT extend beyond therapy sessions in patients with ABI undergoing inpatient neurorehabilitation. We hypothesized that patients receiving AAT would demonstrate greater improvements in socioemotional skills compared to those receiving standard care.

**Methods:**

Over 6 weeks, participants received two weekly sessions of occupational, speech, or physiotherapy with or without animals. The primary outcomes were social behavior and positive and negative emotions assessed via behavioral analyses in a standardized social situation. The secondary outcomes included depression, social cognition, therapy motivation, quality of life, and therapy adherence. The outcomes were evaluated at baseline, post-intervention, and 6- and 12-week follow-ups. Due to substantial attrition, the preregistered primary endpoint at 12-week follow-up could not be analyzed as planned. Therefore, analyses focused on post-assessment outcomes and were exploratory. Linear regression models were used to estimate mean differences.

**Results:**

Seventy patients with ABI (*M* = 51.04 years, *SD* = 13.39; 27.14% female) were randomly assigned to the AAT intervention group (*n* = 43) and the treatment-as-usual (TAU) control group (*n* = 27). In exploratory analyses at post-assessment, the AAT group showed higher social behavior than the TAU group (*difference* = 3.24; 95% CI [0.87, 5.62]; *p* = .009). No significant group effects were found for the other outcomes.

**Conclusion:**

Given the exploratory analyses following protocol deviation, these findings provide preliminary evidence that AAT may improve social behavior beyond therapy sessions in adult patients with ABI undergoing inpatient rehabilitation. Further studies are needed to evaluate the durability and real-world relevance of this effect.

**Clinical Trial Registration:**

ClinicalTrials.gov, identifier NCT03687671.

## 1 Introduction

Patients with acquired brain injury (ABI) frequently experience substantial impairments of physical, emotional, cognitive, and social functioning that restrict their ability to perform daily tasks or engage in social interactions, often impacting their participation in rehabilitation (1, 2). Traditional therapies for ABIs emphasize long-term, multimodal rehabilitation of cognitive, social, physical, and psychological functioning (3). They address the most common behavioral complications, such as changes in mood, disinhibition, and executive dysfunction (4). For instance, changes in mood, such as apathy, may be characterized by a lack of motivation (5)—a critical factor for therapy success in patients with ABI (6). Furthermore, executive dysfunction can impair a patient's ability to organize, plan, execute, or conceptualize tasks, or to maintain mental flexibility (5). Those challenges significantly affect patients’ commitment and progress in ABI therapy.

Another serious problem for patients with ABI is social dysfunction (7, 8). People with traumatic brain injuries may suffer from altered affective responsivity, which can lead to reduced empathy, difficulties recognizing emotions, and impaired emotional expression (9, 10). Moreover, patients with brain injuries can have reduced social perception skills, difficulties in understanding the intentions of others, and social communication problems with impairments in social language (11, 12). Furthermore, disinhibition is linked to impaired social adaptation, such as violating norms and conventions (13). Reduced physical and cognitive capacity, along with impaired self-regulation, may lead patients with ABI to withdraw from social relationships and experience reduced social connectedness (14)—another important predictor of rehabilitation outcomes in patients with ABI.

Coping with the physical, cognitive, and social changes may result in frustration, isolation, hopelessness, and psychological distress (15). This may cause psychological problems and reduce patients’ quality of life (7, 16). The most common psychological problem for patients with ABI is depression (16), which adversely impacts rehabilitation motivation, resulting in skipped therapy sessions and persistent physical impairments (17). Moreover, social isolation is associated with faster cognitive decline and a higher risk of mortality (18). Targeting the socioemotional functioning of patients with ABI is therefore essential both to decreasing the risks of behavioral disorders and psychiatric comorbidity and to enhancing quality of life through rehabilitation (1, 14).

Animal-assisted therapy (AAT) has been increasingly recognized for its potential to enhance psychosocial functioning among patients with ABI in rehabilitation (19, 20). While AAT targets improvements in social, emotional, cognitive, and physical health (21), research has largely focused on its physical benefits. Reviews on the effectiveness of AAT across various neurological disorders have consistently reported enhancements in physical fitness, such as cardiovascular health and increased activity levels, and motor performance, including reduced limb spasticity and better balance (20, 22, 23). However, studies have shown that AAT can also support socioemotional and neuropsychological processes in patients with various neurological disorders, significantly enhancing their recovery across various domains (24–26).

AAT likely combines animal-specific components (e.g., tactile interaction, movement-based engagement, and relational qualities) with non-specific therapeutic factors such as therapist attention, social interaction, novelty, and treatment expectancy (27). Importantly, the human capacity to form meaningful social bonds is not limited to human–human interactions but extends to relationships with animals, reflecting a shared evolutionary history and neurobiological mechanisms underlying attachment and social behavior (28–30). Through processes such as attachment formation, stress modulation, and emotional support, animals may create a sense of safety and trust that facilitates social engagement (31–33). In addition, animals can act as social catalysts, promoting interaction and communication between patients and others, thereby indirectly supporting human–human social functioning (34–36). In this context, the animal is not merely a passive stimulus but an active component of the therapeutic process, serving as a social partner that elicits interaction, provides multisensory stimulation, and is integrated into goal-directed therapeutic tasks, thereby supporting both socioemotional and functional rehabilitation processes (37, 38).

Consistent with these mechanisms, studies have suggested that AAT can enhance neuropsychological functioning in patients with ABI by increasing concentration and alertness (39), attention (40, 41), and memory retention (42). These effects appear to be linked to heightened prefrontal brain activity and emotional engagement during interactions with animals (43). Furthermore, patients with ABI had a better mood and more positive emotions in the presence of animals during therapy sessions compared to similar therapy sessions without an animal present, leading to higher engagement in the therapy process (44). They also reported feeling more secure, accepted, comforted, at ease, and motivated when an animal was present (45). During therapy sessions, AAT may facilitate recovering psychological functioning (17) and reaching the treatment goals of relaxation and contentment (46). It also effectively alleviates disease symptoms, which translates into tangible benefits in daily life, such as enhanced happiness, self-worth, quality of life, and general well-being (23, 25). Further, children with severe neurological impairments have experienced a lot of fun during AAT (46, 47), engaged more with others, and adhered better to group norms (47). In other studies, the positive experiences associated with animal interactions have led to more favorable perceptions of therapy, greater treatment motivation and satisfaction, and a higher commitment to the therapeutic process (42, 45). In addition, patients involved in AAT exhibited marked improvements in social involvement and competence, characterized by increased smiles, laughter, and enhanced verbal and nonverbal communication during therapy when an animal was present compared to when no animal was present (44).

Overall, the integration of AAT into rehabilitation programs seems to be a promising approach for patients with ABI. However, most of these positive effects have only been observed during the presence of the animal in the therapy sessions (41), and it is unclear to what degree these effects translate into situations outside therapy sessions. To address this gap, the present randomized controlled trial aimed to investigate whether AAT improves socioemotional functioning and whether its effects extend to a standardized social situation outside therapy sessions in patients with ABI undergoing inpatient neurorehabilitation. Patients’ socioemotional skills were assessed at baseline, after 6 weeks of therapy, and at two follow-up assessments. We hypothesized that socioemotional skills would increase more in patients with ABI treated with AAT than in patients treated with treatment as usual (TAU). Furthermore, we hypothesized that changes in patients’ depressive symptom severity, social cognition, therapy motivation, and quality of life would be higher after AAT and that patients with ABI would show higher therapy adherence with AAT compared to TAU.

## 2 Methods

### 2.1 Participants

We recruited 70 patients in inpatient neurorehabilitation for an ABI from either traumatic (TBI) or nontraumatic (non-TBI) causes. Eligibility for participation was determined on the following inclusion criteria: 1) age of 18 years or older, 2) diagnosis of an ABI, 3) a Functional Independence Measure (FIM) score exceeding 55 points, and 4) informed consent, confirmed through a signature by the patient or their legal representative. Exclusion criteria were: 1) any potential conflict of interest involving the investigator's immediate family, employees, or dependents and 2) medical contraindications for contact with animals such as an allergy, phobia, etc. The study ensured continuous monitoring of potential risks, including whether an animal harmed a patient, whether a patient harmed an animal, or whether a significant medication adjustment during the study period affected a patient's stability. Any of these events would have led to exclusion from participation in the study. Ethical approval was granted by the Ethics Commission of Northwestern and Central Switzerland (EKNZ) under project ID 2018-0130. The study protocol adhered to the Declaration of Helsinki and Swiss law and regulatory requirements. In addition, AAT was performed according to the IAHAIO (International Association of Human-Animal Interaction Organizations) guidelines (21). The study was registered on ClinicalTrials.gov (identification number NCT03687671, registration date September 27, 2018) and received approval for protocols involving animals from the Veterinary Office of the Canton of Basel-Stadt (29573/2713) and the Canton of Basel-Land (BL/552/2023/35940). All research methods complied with the relevant ethical and regulatory guidelines, and we adhered to the CONSORT 2025 reporting guidelines. One adverse event related to the study was reported: One patient had an allergic reaction to cat hair. To prevent further incidents, the patient ended the therapy sessions early and dropped out of the study.

### 2.2 Study design and procedure

**Figure 1:**
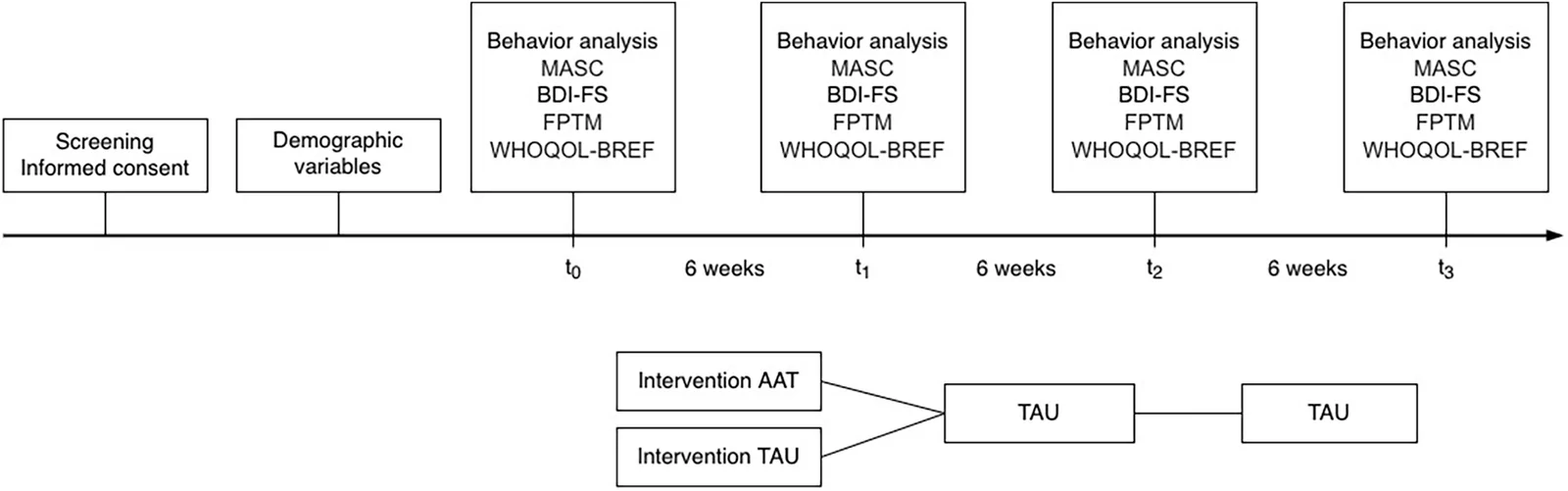
Timeline of the study design, including assessment points and intervention phases. AAT, animal-assisted therapy; BDI-FS, Beck Depression Inventory Fast Screen; FPTM, psychotherapy motivation questionnaire (German: Fragebogen zur Psychotherapiemotivation); MASC, Movie for the Assessment of Social Cognition; TAU, treatment as usual; t_0_, baseline; t_1_, post-assessment; t_2_, follow-up I; t_3_, follow-up II; WHOQOL-BREF, World Health Organization Quality of Life-BREF.

This single-center randomized controlled trial with repeated measurement was conducted at REHAB Basel, a neurorehabilitation and paraplegia clinic in Switzerland, between November 1, 2018, and June 20, 2024. Participants were randomly assigned to either the AAT intervention group, which received animal-assisted occupational, speech, or physiotherapy for 6 weeks, or the TAU control group, which received the equivalent therapy without animals. Data were collected at four time points spaced 6 weeks apart over 18 weeks: baseline before the intervention (t_0_), post-assessment immediately after the 6-week treatment (t_1_), follow-up I at 12 weeks (t_2_), and follow-up II at 18 weeks (t_3_). After the 6-week intervention, all participants received TAU. Each measurement point was divided into two sessions: The first included a video-based social cognition task and questionnaire; the second involved behavioral coding of a standardized social interaction and three self-report questionnaires on depressive symptoms, therapy motivation, and quality of life. Assistance (e.g., reading questions aloud) was provided as needed. The research staff included licensed psychologists (EP, SD, CH, AH, and KH), master's students in clinical psychology, and research interns. Due to the nature of AAT, blinding was not possible. Figure 1 illustrates the timeline of the study design.

### 2.3 Recruitment and randomization

Seventy adult patients with an ABI who were undergoing inpatient neurorehabilitation at REHAB Basel were recruited through flyers and direct communication by therapists or physicians. Patients were proposed as eligible by a treating physician or therapist. They were then screened for inclusion and exclusion criteria. If they were still eligible, they received detailed information about the study's purpose, procedure, expected duration, and potential risks and benefits. They were informed that participation in the study was voluntary and that they could withdraw at any time without affecting further medical assistance and treatment. They were further informed that obtained data would be handled strictly confidentially and only designated research staff would have access to the collected data. After written informed consent was obtained, the research staff enrolled the participants. Using a computer-generated random sequence in Microsoft Excel, participants were randomly assigned by the research staff to either the AAT or the TAU group. All the study staff had access to the generated sequence. The formula used did not enforce balanced allocation, which led to imbalanced group sizes. *Post hoc* tests revealed that the generated random sequence had a 1.6:1 ratio favoring the AAT group (43 patients in the AAT group vs. 27 patients in the TAU group).

### 2.4 Intervention

Over 6 weeks, participants received two occupational, speech, or physiotherapy sessions per week, leading to 12 sessions in total. In the AAT group, the sessions incorporated animals, whereas the TAU group had conventional therapy sessions. Each therapy session lasted approximately 45 minutes. The same therapist planned and conducted the interventions for both conditions to control for therapist-related effects. To ensure a balance between standardization and individualization, therapists selected session activities from a predefined collection of tasks developed in collaboration with the interdisciplinary therapy team. Based on this collection, therapists determined the content and format of each session, allowing for a high degree of comparability across conditions while still addressing individual rehabilitation goals. Tasks were designed to be performed in both groups, with comparable structures but different materials. For example, patients prepared food for an animal (AAT) or a fruit salad for humans (TAU), or completed structured activities such as building and navigating an obstacle course together with a therapy animal (AAT) or a ball (TAU).

#### 2.4.1 Animal-assisted therapy (AAT)

**Figure 2:**
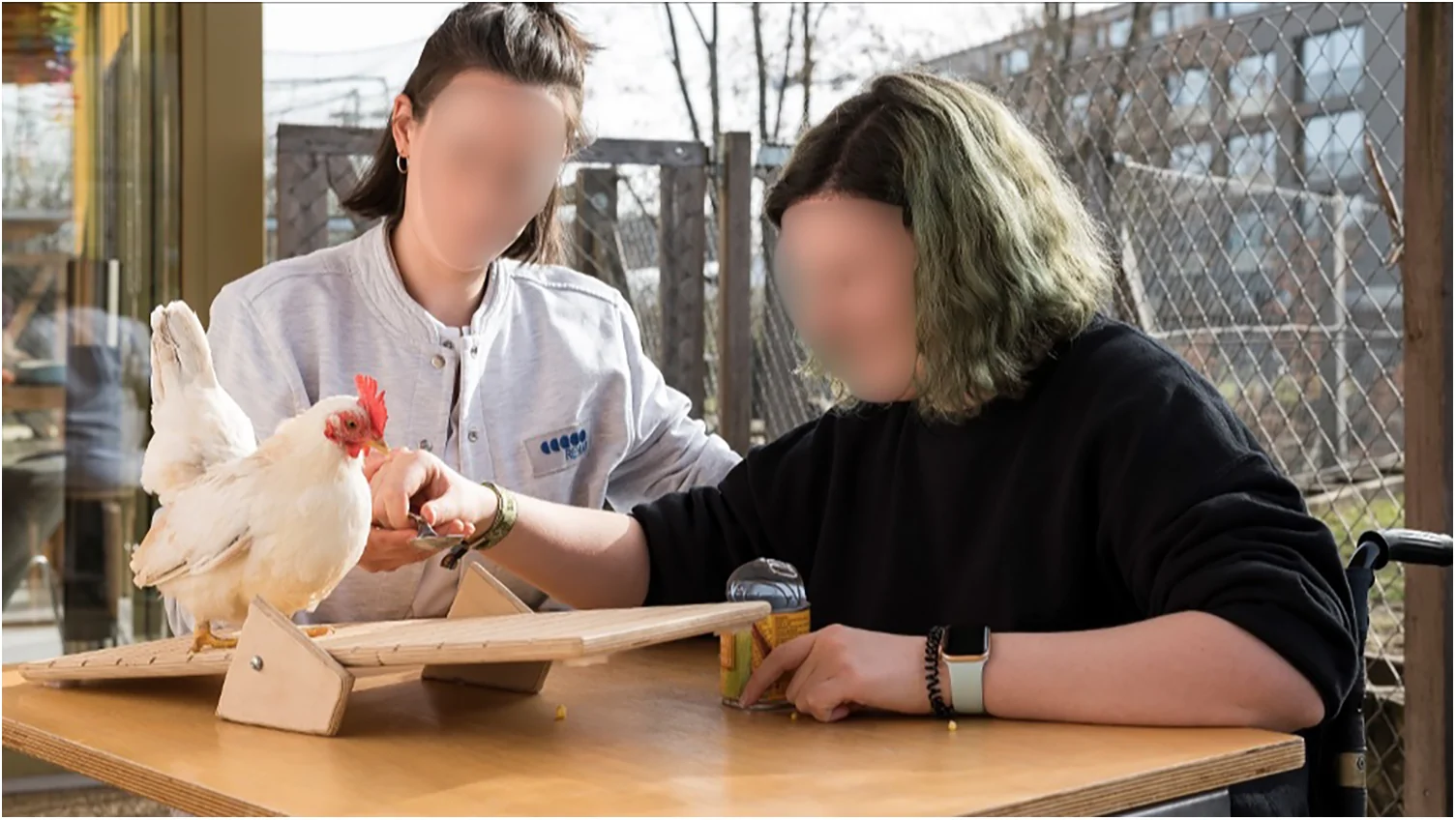
Example of an animal-assisted occupational therapy session involving a food-guided exercise course with a chicken in a specialized room of the therapy garden.

**Figure 3:**
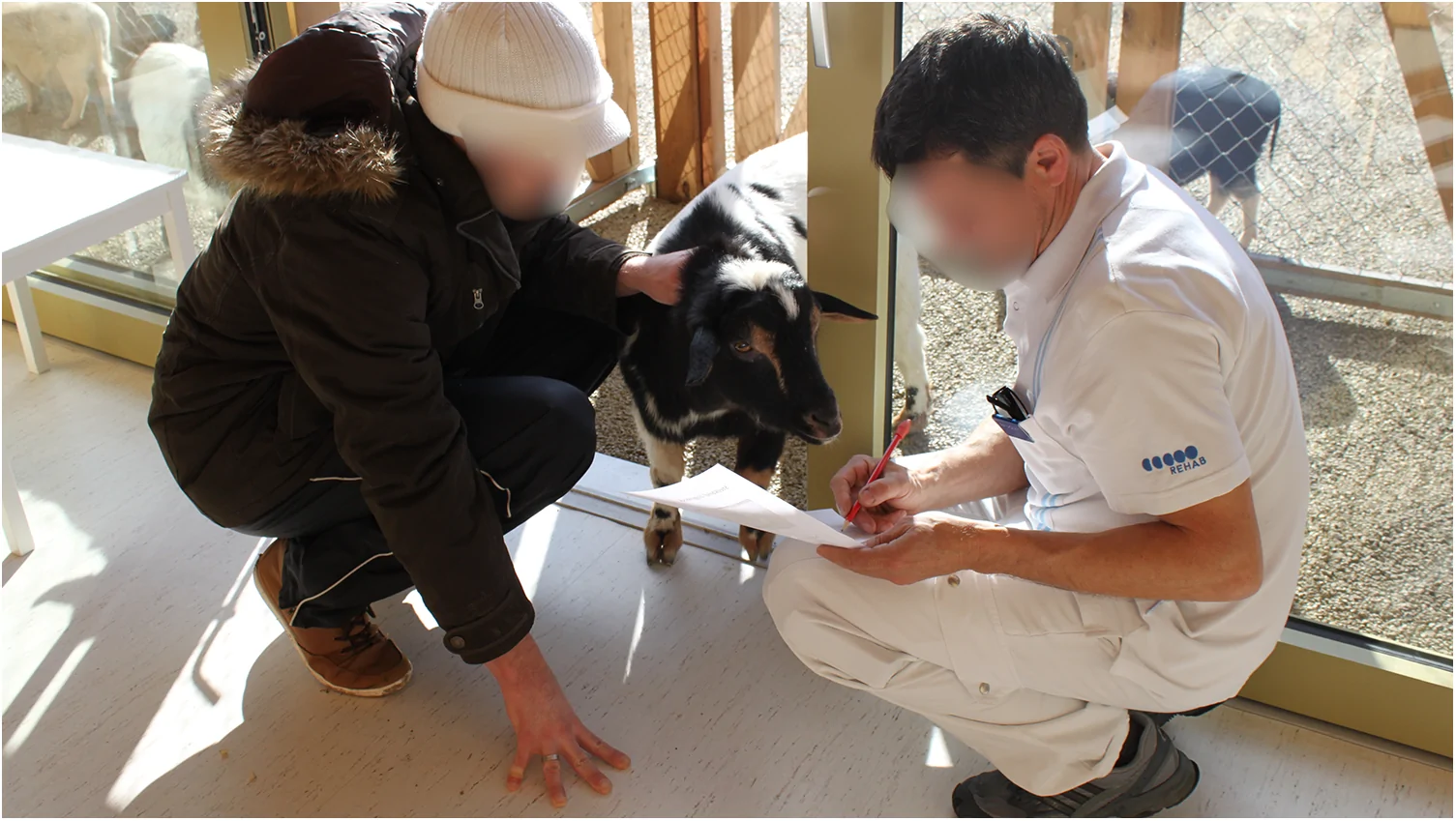
Example of an animal-assisted speech therapy session involving reading and writing exercises with a goat in a specialized room of the therapy garden.

**Figure 4:**
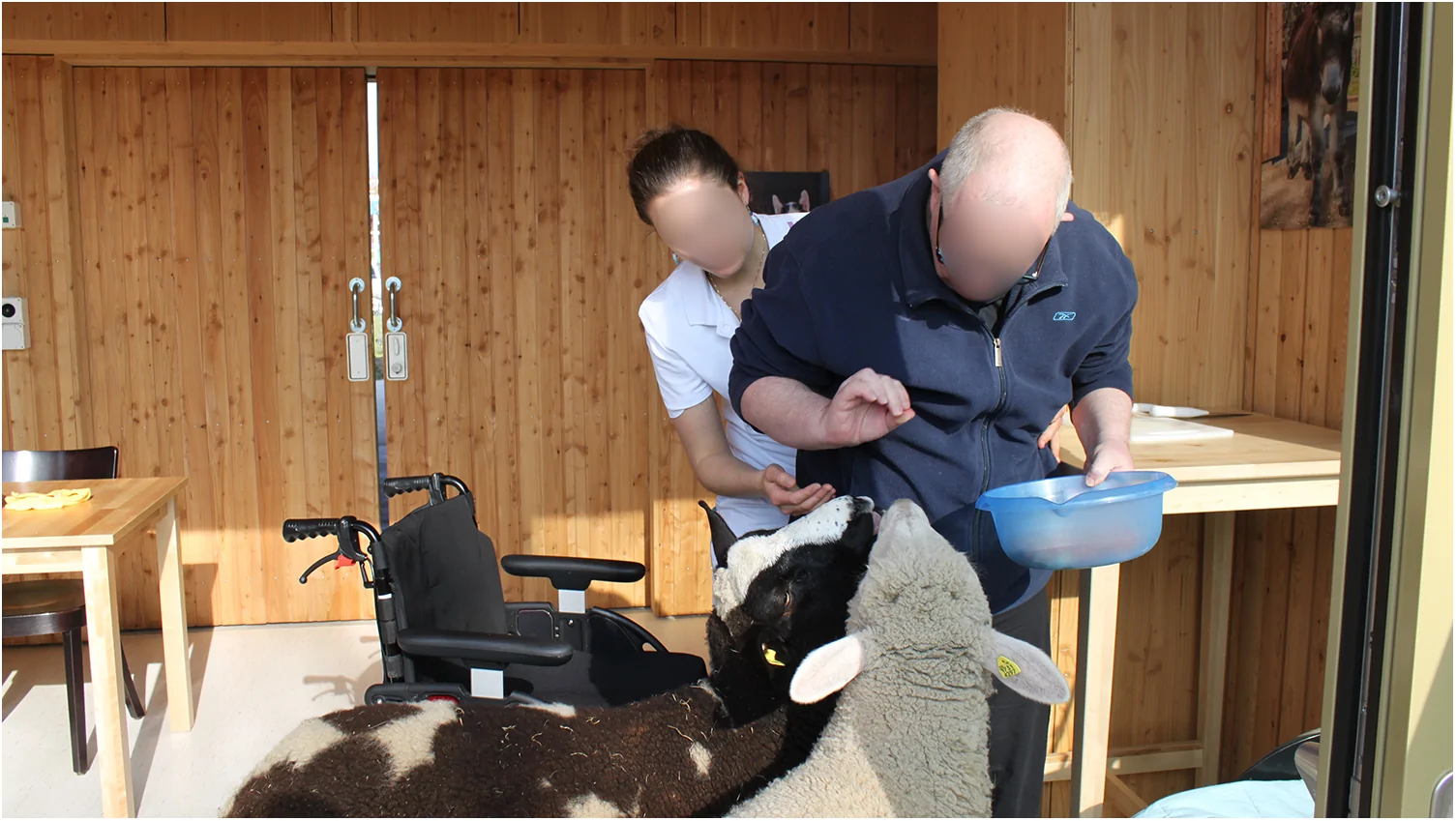
Example of an animal-assisted physiotherapy session involving walking and feeding activities with sheep in a specialized room of the therapy garden.

The AAT sessions were held in specially equipped rooms or the therapy garden. If patients were sufficiently mobile, sessions took place in the therapy garden, which included outdoor areas and specially equipped indoor spaces accessible to animals. The therapy garden was located on the clinic grounds and was accessible to patients on foot, in a wheelchair, or in a bed. It also featured a designated area for horses, where patients could interact with them directly or have the horses brought to accessible locations. For patients with limited mobility or medical restrictions, smaller animals were brought into specially equipped rooms within the clinic building. The therapy sessions were conducted by licensed occupational therapists, speech therapists, or physiotherapists with training in AAT and facilitated by animal handlers. During each session, the AAT therapist ensured the patient's safety while an animal handler cared for the animal's welfare. The therapists facilitated interactions between the patients and the animals, promoting physical contact if possible, as physical contact is considered essential for building a human–animal bond (31, 34), and tailored the setting and materials to each patient's therapeutic goals. Figures 2–4 show examples of therapeutic settings.

The animals were selected for AAT based on each patient's individual rehabilitation goals, the therapist's clinical assessment, and the animal's availability, reflecting a flexible and patient-centered approach. For example, larger animals (e.g., horses, goats, or sheep) were often included for patients working on gross motor skills such as balance, posture, or mobility, whereas smaller animals (e.g., guinea pigs, rabbits, or chickens) were selected for patients focusing on fine motor skills such as grasping, coordination, or gentle handling. In addition, patients’ individual preferences were taken into account, including previous experiences with animals (e.g., pet ownership), cultural considerations (e.g., avoiding certain species), and personal affinity for specific animals, to enhance engagement and the therapeutic alliance. This selection strategy aligns with current evidence indicating that AAT effectiveness in adult neurorehabilitation is primarily driven by the quality of the human–animal relationship and goal-directed interaction rather than by the specific animal species (48, 49), with species acting as an individual-level moderator rather than as the primary driver of therapeutic outcomes (50, 51).

The animals included chickens, guinea pigs, rabbits, cats, dogs, minipigs, sheep, goats, horses, birds, and a mule. All the involved animals were trained for AAT, were accustomed to working with patients with ABI, and received regular veterinary care. Strict hygiene practices, including hand washing before and after sessions, were implemented to prevent zoonosis. At the beginning of each AAT session, the emotional status of the animal involved was assessed to determine its fitness to interact with the patient. This assessment was conducted by trained animal handlers who had established close relationships with the animals and were familiar with species-specific as well as individual behavioral signals. The evaluation was based on the observation of behavioral indicators, such as body posture, ear, tail, or wing position, as well as behaviors including hiding, licking, panting, or vocalizations (e.g., chirping), which may signal discomfort or stress. Importantly, all the animals were allowed to interact freely with the patients whenever possible and to withdraw from activities at any point during the therapy sessions, ensuring their comfort and safety.

#### 2.4.2 Treatment as usual (TAU)

The TAU sessions mirrored the AAT sessions in content and setting. The therapists selected the content and format of the sessions from the predefined collection of tasks, resulting in a high level of comparability between the two conditions. Similarly to the AAT group, the therapists thoroughly assessed each patient's therapeutic goals to tailor the sessions accordingly. Each TAU session that corresponded to an AAT session was conducted by the same licensed occupational therapist, speech therapist, or physiotherapist.

### 2.5 Measurements

#### 2.5.1 Behavior analyses

To assess transfer effects beyond therapy sessions, socioemotional skills were evaluated in a standardized social situation designed to approximate real-life social interactions. All patients participated in a structured 10-minute activity, which was videotaped for analysis of their socioemotional skills. The activity involved building a tower with wooden construction planks (KAPLA® planks) together with two members of the research staff, who interacted with the patients in a highly standardized way to provoke specific reactions. The selection of this task was informed by clinical expertise and developed in an iterative process involving therapists, nurses, physicians, and researchers experienced in the rehabilitation of patients with ABI, with the aim of eliciting a broad range of socially relevant behaviors within a controlled yet interactive setting. Using Noldus Observer software (versions XT 12 and XT 16) for video analysis, every second of the interaction was coded for the presence or absence of defined behaviors. The coding involved detailed monitoring for both state and count behavior variables. State behaviors were quantified by marking each occurrence and calculating the percentage and duration in seconds of each behavior relative to the session length. Count variables captured the frequency of specific behaviors per session and were quantified in rates per minute. Adjustments were made for periods when faces were not observable, such as during mask wearing during the COVID-19 pandemic. The variables were calculated relative to the total observable duration.

The predefined ethogram used for coding was developed based on established systems (43, 44) to ensure high comparability, and was further refined in an iterative process with clinical experts working with patients with ABI to ensure relevance to this population. The ethogram encompassed the following behavior categories: 1) patients’ verbal behaviors like active vocalizations, reactive vocalizations, questions directed at the research staff, and nondirected vocalizations; 2) patients’ nonverbal behaviors related to communication like eye contact and body movements; 3) patients’ emotions like astonishment, sadness, anger, fear, neutral states, and positive emotions; 4) patients’ prosocial behaviors like offering water, engaging in conversation, or including research staff in the game; 5) research staff's behavior including verbal and nonverbal behavior as described for patients, and positive emotions; and 6) event variables including incidents like planks falling, water being offered, or the tower crashing (see Supplementary Table 1 for the comprehensive ethogram). These categories were selected to capture multiple dimensions of social functioning (e.g., verbal, nonverbal, emotional, and prosocial behavior), reflecting the heterogeneous nature of socioemotional impairments following ABI. To ensure reliability, coders were trained in advance by coding the same training video. They were then required to achieve a Cohen's kappa coefficient (*k*) of over .80, confirming high interrater reliability, before they proceeded with the coding tasks.

#### 2.5.2 Primary outcomes: socioemotional skills

Our primary outcomes were the three socioemotional variables: social behavior, positive emotions, and negative emotions. Social behavior encompassed the relative duration of verbal behavior, nonverbal behavior, and prosocial interactions and communications between the patient and the research staff, all measured via behavior analysis. Verbal behavior was categorized as active, reactive, active questions, or undefined. Active verbal behavior was initiated by the patient and directed toward the research staff. Reactive verbal behavior was defined as responses to cues from the research staff, and active questions were when patients directed a question at the research staff. Undefined verbal behavior included nontargeted speech acts, such as mumbling or self-referential statements. Nonverbal behavior included eye contact and body movements directed toward the research staff, while prosocial behavior involved assisting them with tasks such as pouring water and handing over wooden planks. Nonverbal behavior variables could be coded in parallel. Positive emotions were coded when a patient smiled or lifted the corners of their mouth. Negative emotions comprised the mutually exclusive variables of astonishment, sadness, anger, and fear. Each behavioral category or subcategory quantified the percentage of time spent displaying each type of behavior during the standardized social interaction.

### 2.6 Secondary outcomes

#### 2.6.1 Depressive symptom severity

The German version of the Beck Depression Inventory Fast Screen (BDI-FS) (52) was administered to assess depressive symptom severity. This self-administered instrument includes seven items targeting the key symptoms of sadness, pessimism, feelings of failure, loss of joy, withdrawal, self-reproach, and suicidal thoughts. Its fast application reduces patient burden, and the questionnaire was specifically designed for individuals with physical health impairments (53). Participants rate each item on a 4-point Likert scale, ranging from 0 (*no symptom presence*) to 3 (*high symptom severity*), yielding a total possible sum score of 21 points, with higher scores indicating higher depressive symptom severity. It classifies depression severity as follows: scores of 0–3 indicate minimal depression, 4–8 mild depression, 9–12 moderate depression, and 13–21 severe depression (54). The BDI-FS demonstrates high internal consistency, with a Cronbach's alpha (*α*) = .84, confirming its reliability in measuring depression (55). Furthermore, its strong external validity has been supported by successful validations across diverse populations and settings (54).

#### 2.6.2 Social cognition

The German version of the Movie for the Assessment of Social Cognition (MASC) (56) was used to evaluate the patients’ social cognition, namely, mentalizing abilities such as theory of mind (ToM) or the ability to attribute mental states to others and to predict their behavior. The test consists of a 15-minute film depicting four characters at a dinner party. The film explores their interactions, which vary from simple two-person exchanges to more complex four-person dynamics and showcases a range of emotions and social complexities such as friendship and romantic interest. The characters’ diverse motives and personalities lead to situations involving traditional concepts and challenges of social cognition like first- and second-order false beliefs, deception, faux pas, persuasion, metaphors, sarcasm, or irony. The entire assessment requires about 45 minutes to complete. The film is stopped 43 times during its course for patients to answer questions on the characters’ mental states, the conversational content, and control questions to test memory and understanding. The assessment includes 51 multiple-choice items, categorized to measure emotions (17 items), thoughts (7 items), intentions (18 items), verbal interactions (19 items), nonverbal cues (16 items), and six control questions. Single items may cover more than one domain.

The MASC assessment uses a scoring system where correct responses earn 1 point and incorrect ones 0 points, leading to a possible total score between 0 and 51. Higher scores indicate better ToM abilities. Additionally, the assessment includes a qualitative-error analysis that categorizes incorrect responses into three subcomponents: (a) less ToM, indicating insufficient mental-state reasoning; (b) no ToM, where responses show a total absence of mental-state attributions; and (c) excessive ToM, characterized by overinterpretation of mental states in situations where none are applicable. This approach quantifies ToM capabilities and pinpoints specific cognitive challenges in social interactions such as deficits in emotion recognition (57). The MASC demonstrates robust internal consistency between *α* = .82 and *α* = .84 (56). Although the MASC has been primarily validated in psychiatric populations, including those with autism-spectrum disorder (58), paranoid schizophrenia (57), borderline and narcissistic personality disorder (58, 59), and has also been applied in depressive disorders (60), its validation in patients with ABI remains limited. Nevertheless, the MASC was selected due to its high ecological validity and its ability to assess complex, dynamic aspects of social cognition, which are relevant to social-cognitive impairments observed following ABI.

#### 2.6.3 Therapy motivation

Therapy motivation was assessed using the Fragebogen zur Psychotherapiemotivation (FPTM), a German self-report questionnaire (61). Designed for individuals aged 16 and older, the FPTM includes 39 items with four response options for each item on a 4-point Likert scale ranging from 1 (*strongly disagree*) to 4 (*strongly agree*). The FPTM measures the six subscales psychological distress (10 items; range: 10–40), hope for improvement (seven items; range: 7–28), denial of psychological need for help (seven items; range: 7–28), knowledge of psychotherapy (five items; range: 5–20), initiative in seeking treatment (four items; range: 4–16), and symptom-related care by others (six items; range: 6–24). For each subscale, a mean is calculated; higher values indicate higher therapy motivation, except for the subscales denial and symptom-related care, where higher values indicate lower therapy motivation (62). Completing the questionnaire takes approximately 10 minutes. The FPTM has been validated and normed in various psychosomatic rehabilitation clinics, demonstrating internal consistency with Cronbach's alphas ranging between *α* = .68 and *α* = .92 (63). The FPTM has been used in evaluation studies and as part of routine diagnostics in psychosomatic or psychotherapeutic specialist clinics with patients with a broad range of disorders (64).

#### 2.6.4 Quality of life

Quality of life was measured using the World Health Organization Quality of Life-BREF (WHOQOL-BREF), a concise yet comprehensive self-report comprising 26 items, each of which is rated on a 5-point Likert scale and ranges from 1 (*not at all*/*very poor*/*very dissatisfied*/*never*) to 5 (*very good*/*an extreme amount*/*extremely*/*always*) (65). The questionnaire is designed to be completed in approximately 5–10 minutes. The WHOQOL-BREF enables the creation of a quality-of-life profile across four domains—physical health (seven items; range: 7–35), psychological well-being (six items; range: 6–30), social relationships (three items; range: 3–15), and the environment (eight items; range: 8–40)—along with two global items assessing overall quality of life and general health status (range: 2–10). For each domain, a mean is calculated. Then, the mean scores are converted to a scale from 0 to 100 to facilitate comparisons across domains with different numbers of items, with high values indicating a high quality of life. The questionnaire shows robust internal consistency, with a Cronbach's alpha for its subscales ranging from *α* = .57 to *α* = .88 (66). Notably, the WHOQOL-BREF effectively discriminates between individuals with health impairments and those without, and it distinguishes between physical and psychological illnesses, thereby validating its utility across varied health contexts (67).

#### 2.6.5 Therapy adherence

Treatment adherence was measured as the number of therapy sessions attended by patients in both conditions, calculated as the number of sessions actually attended out of the 12 planned sessions. Intervention completion was operationalized as attendance at a minimum of eight sessions, corresponding to two-thirds of the planned intervention.

### 2.7 Statistical analyses

#### 2.7.1 Calculation of sample size

An *a priori* power analysis indicated that a total sample size of *N* = 70 (two groups) would be sufficient to detect a medium effect size (Cohen’s *d* = 0.60) with 80% power at a two-sided significance level of *α* = .05, and a large effect (*d* = 0.80) with 95% power.

#### 2.7.2 Data processing

Social behavior was computed as the sum of the variables active vocalization, reactive vocalization, active question, nondirected vocalization, body movement, eye contact, and prosocial behavior, divided by the product of the number of categories and variables, so as to account for parallel behavior coding and to ensure that they could maximally add up to 100%: social behavior = ([*active vocalization* + *reactive vocalization* + *active question* + *nondirected vocalization*] + [*body movement* + *eye contact*] + [*prosocial behavior*])/3*7. This weighting was based on clinical experience, as described in Hediger and colleagues (44), and accounts for the fact that patients with ABI may exhibit social behavior differently depending on their specific impairments. Accordingly, each mode of social behavior was considered equally relevant.

We also computed a total percentage score for the variable negative emotions by calculating the mean of the variables astonishment, sadness, anger, and fear: negative emotions = (*astonishment* + *sadness* + *anger* + *fear*)/4. Furthermore, we computed the variable social behavior of research staff by calculating a sum score for the variables active vocalization, reactive vocalization, active question, nondirected vocalization, body movement, and eye contact coded for research staff. We further calculated a ratio between patient and research staff behavior ([*total duration behavior patient*/*total duration behavior research staff*] × 100) for the variables social behavior and positive emotions, accounting for potential research staff effects on the primary outcomes (44). However, in our sample, we did not find any group differences, so this ratio was only explored descriptively.

#### 2.7.3 Primary and secondary analyses

Due to substantial attrition after post-assessment (t_1_), a full intention-to-treat (ITT) analysis and the preregistered primary endpoint at 18 weeks (t_3_) could not be meaningfully evaluated. Consequently, all primary and secondary analyses followed a modified intention-to-treat (mITT) approach (68) that included all the randomized participants who completed at least one measurement at (t_1_). Because the high drop-out rate beyond (t_1_) substantially limited statistical power, longitudinal linear mixed-effects models across later time points were not feasible. For that reason, we conducted exploratory analyses focusing on post-assessment outcomes (t_1_) and reported these analyses as representing a deviation from the original analysis protocol, which planned confirmatory longitudinal mixed-effects analyses to test treatment effects at follow-up II (t_3_).

First, we explored baseline (t_0_) differences for all the outcome variables, comparing study completers (data at t_1_) with non-completers (no data at t_1_) using two regression models: an unadjusted model that only included completion status and an adjusted model that additionally accounted for the treatment condition. Then, we calculated linear regression models (Model 1) for all the outcome variables, including treatment condition (TAU vs. AAT) as a fixed independent variable and the corresponding baseline values as covariates (ANCOVA approach). Because only one post-intervention observation per participant was analyzed, no random effects were specified. The model assumptions for linear-regression models and the normal distribution of the dependent variable were visually checked with QQ plots, histograms, and boxplots, focusing on linearity, variance homogeneity, and the normal distribution of residuals.

Descriptive results are reported in Tables 1, 2 as means and standard deviations for each condition and measurement time. Effect estimates (*difference*), *p*-value, and the 95% confidence interval (CI) are presented in Table 3. All statistical analyses were conducted using R (versions 2024.12.1 + 563 and 2026.01.0 + 392) (69) with the packages broom (70), broom.mixed (71), dplyr (72), ggplot2 (73), lme4 (74), openxlsx (75), purrr (76), readr (77), and tidyr (78). The significance level was set at *p* ≤ .05.

**Table 1:** Sample characteristics of the randomized study participants (*N* = 70) and intervention characteristics.

Variables	Level	AAT (*n* = 43)		TAU (*n* = 27)	
**Age** (*M*, *SD*)		46.64	12.69	60.35	9.75
**Gender** (*n*, %)	Female	10	23.26	9	33.33
	Male	33	76.74	18	66.67
**Acquired brain injury** (*n*, %)	Traumatic	11	25.58	3	11.11
	Hemorrhage	14	32.56	11	40.74
	Both	9	20.93	4	14.81
	Other forms of non-traumatic brain injury	9	20.93	9	33.33
**Days since DOI** (*M*, *SD*)		123.35	66.24	179.41	286.41
**FIM** (*M*, *SD*)		89.73	16.90	87.87	16.30
**No. of sessions attended** (*M*, *SD*)		9.71	3.28	10.47	2.85
**Therapy modality** (*M*, *SD*)	Occupational therapy	5.62	3.95	7.53	4.10
	Physiotherapy	2.24	3.33	2.07	3.15
	Speech therapy	1.39	2.81	0.80	2.83
**Animal species** (*n*, %)	Dogs	6	6.12	*NA*	*NA*
	Cats	4	4.08	*NA*	*NA*
	Guinea pigs	15	15.31	*NA*	*NA*
	Minipigs	9	9.18	*NA*	*NA*
	Horses	15	15.31	*NA*	*NA*
	Sheep	13	13.27	*NA*	*NA*
	Chicken	9	9.18	*NA*	*NA*
	Goats	10	10.20	*NA*	*NA*
	Rabbits	7	7.14	*NA*	*NA*
	Mule	6	6.12	*NA*	*NA*
	Birds	4	4.08	*NA*	*NA*
**No. of different species per patient** (*n*, %)	1	5	14.29	*NA*	*NA*
	2	9	25.71	*NA*	*NA*
	3	13	37.14	*NA*	*NA*
	4	5	14.29	*NA*	*NA*
	5	2	5.71	*NA*	*NA*
	6	1	2.86	*NA*	*NA*

AAT, animal-assisted therapy; DOI, date of injury; FIM, Functional Independence Measure; TAU, treatment as usual. Demographic and clinical characteristics are reported for all randomized participants. Animal-related variables are reported only for AAT participants with available animal-contact records (*n* = 35), irrespective of whether they met the minimum intervention dose criterion (≥8 sessions). Percentages for animal-related variables are based on this subgroup.

**Table 2:** Descriptive statistics for the primary and secondary outcomes across assessment timepoints.

Outcomes	t_0_						t_1_						t_2_						t_3_					
	AAT			TAU			AAT			TAU			AAT			TAU			AAT			TAU		
	*n*	*M*	*SD*	*n*	*M*	*SD*	*n*	*M*	*SD*	*n*	*M*	*SD*	*n*	*M*	*SD*	*n*	*M*	*SD*	*n*	*M*	*SD*	*n*	*M*	*SD*
**Behavior analyses**																								
Social behavior	35	38.33	2.41	12	38.98	2.82	31	40.19	4.31	13	37.85	2.45	26	39.35	3.64	8	37.80	1.59	19	38.51	2.76	7	39.21	3.14
Positive emotions	35	10.54	11.48	12	16.30	23.61	31	9.59	8.22	13	12.56	11.64	26	11.58	11.15	8	12.47	11.40	19	15.34	18.11	7	15.91	11.79
Negative emotions	35	0.09	0.19	12	0.04	0.12	31	0.17	0.50	13	0.03	0.07	26	0.04	0.09	8	0.04	0.09	19	0.07	0.11	7	0.04	0.09
**BDI-FS**																								
	38	2.68	3.31	18	2.72	2.30	29	2.00	2.59	12	1.33	1.61	26	1.88	2.23	8	2.13	2.10	20	1.10	1.29	7	0.86	0.90
**MASC**																								
	35	23.97	7.68	18	21.61	8.22	26	27.58	7.68	11	25.00	5.33	22	27.41	7.85	8	26.50	4.99	17	29.12	6.73	7	28.43	3.46
**FPTM**																								
Psychological distress	34	1.91	0.73	15	1.97	0.57	28	1.75	0.76	11	1.49	0.43	26	1.81	0.79	8	1.59	0.54	18	1.77	0.60	7	1.83	0.50
Hope	35	3.45	0.67	15	3.57	0.45	28	3.70	0.50	11	3.58	0.61	26	3.55	0.59	8	3.59	0.44	18	3.65	0.54	7	3.53	0.46
Denial	35	1.98	0.78	15	2.14	0.87	28	1.75	0.63	11	1.91	0.83	26	1.71	0.61	8	2.09	0.88	18	1.73	0.65	7	1.94	1.05
Knowledge	35	1.67	0.60	15	1.53	0.44	28	1.74	0.81	11	1.76	0.85	26	1.66	0.53	8	1.88	0.89	18	1.82	0.79	7	2.03	0.65
Initiative	35	2.05	1.05	15	1.85	0.91	28	1.76	1.03	11	1.64	0.96	26	1.79	1.14	8	1.53	0.88	18	1.78	1.01	7	1.50	0.46
Symptom-related care	35	2.92	0.73	14	3.19	0.60	28	3.16	0.62	11	3.23	0.54	26	3.17	0.60	8	3.21	0.76	18	3.08	0.63	7	3.31	0.81
**WHOQOL-BREF**																								
Physical health	37	59.52	13.07	17	61.38	7.67	28	60.59	9.50	12	63.10	7.67	25	58.57	9.56	8	63.10	7.67	19	61.59	9.25	7	63.10	8.17
Psychological well-being	37	65.79	13.63	17	65.44	8.43	28	67.80	13.63	12	68.75	8.97	25	70.17	9.45	8	66.67	10.91	19	72.15	7.61	7	69.64	7.50
Social relationships	37	76.35	16.02	17	72.06	17.42	28	75.89	15.77	12	79.51	16.23	25	78.00	16.65	8	84.38	15.06	19	79.61	13.53	7	83.33	9.62
Environment	36	79.25	15.80	16	78.52	9.94	28	79.13	12.09	12	86.24	10.62	25	81.20	11.87	8	85.55	11.20	19	81.91	11.05	7	85.27	10.16

AAT, animal-assisted therapy; BDI-FS, Beck Depression Inventory Fast Screen; FPTM, Psychotherapy Motivation Questionnaire (German: Fragebogen zur Psychotherapiemotivation); MASC, Movie for the Assessment of Social Cognition; TAU, treatment as usual; t₀, baseline; t₁, post-assessment; t₂, follow-up I; t₃, follow-up II; WHOQOL-BREF, World Health Organization Quality of Life-BREF. Descriptive statistics are based on all available observations. Sample sizes vary across outcomes and assessment timepoints due to missing data.

**Table 3:** Linear regression models examining group differences at post-assessment (t₁) (*N* = 44).

Outcomes	Level	Term	*Difference*	95% CI		*p*-value
				*LL*	*UL*	
**Behavior analyses**	Social behavior	Condition	3.24	0.87	5.62	.009*
		Baseline	0.84	0.42	1.26	<.001*
	Positive emotions	Condition	−2.09	−9.07	4.89	.548
		Baseline	0.11	−0.10	0.32	.294
	Negative emotions	Condition	0.16	−0.15	0.48	.299
		Baseline	−0.03	−0.80	0.73	.932
**BDI-FS**		Condition	0.45	−1.17	2.08	.576
		Baseline	0.32	0.03	0.61	.033
**MASC**		Condition	−0.16	−3.50	3.17	.922
		Baseline	0.77	0.56	0.99	<.001*
**FPTM**	Psychological distress	Condition	0.25	−0.14	0.64	.201
		Baseline	0.67	0.42	0.92	<.001*
	Hope	Condition	0.26	−0.08	0.61	.133
		Baseline	0.49	0.23	0.76	<.001*
	Denial	Condition	−0.18	−0.59	0.24	.391
		Baseline	0.64	0.36	0.92	<.001*
	Knowledge	Condition	0.08	−0.49	0.65	.772
		Baseline	0.40	−0.06	0.86	.084
	Initiative	Condition	0.18	−0.44	0.80	.550
		Baseline	0.58	0.30	0.86	<.001*
	Symptom-related care	Condition	0.08	−0.34	0.51	.698
		Baseline	0.40	0.15	0.65	.002*
**WHOQOL-BREF**	Physical Health	Condition	−1.07	−6.30	4.16	.681
		Baseline	0.48	0.26	0.71	<.001*
	Psychological well-being	Condition	1.06	−4.12	6.23	.682
		Baseline	0.46	0.28	0.63	<.001*
	Social relationships	Condition	−5.69	−14.75	3.37	.211
		Baseline	0.65	0.38	0.92	<.001*
	Environment	Condition	−6.19	−13.40	1.01	.090
		Baseline	0.50	0.27	0.74	<.001*
**Therapy adherence**		Condition	−1.01	−2.98	0.97	.310

BDI-FS, Beck Depression Inventory Fast Screen; CI, confidence interval; FPTM, Psychotherapy Motivation Questionnaire (German: Fragebogen zur Psychotherapiemotivation); LL, lower limit of the 95% confidence interval; MASC, Movie for the Assessment of Social Cognition; UL, upper limit of the 95% confidence interval; WHOQOL-BREF, World Health Organization Quality of Life-BREF. The sample comprised all randomized participants who completed at least one outcome assessment at post-assessment (t₁). Outcome-specific sample sizes may vary because of missing data.

* Indicates statistically significant associations (*p* < .05).

#### 2.7.4 Additional analyses

We fitted two additional models to consider relevant context factors. Model 2 included the following relevant covariates for the primary outcomes: age, gender, functional independence, the number of sessions attended, and research-staff behavior during the standardized interaction. For the models of the secondary outcomes, we controlled for the covariates age, gender, functional independence, and number of attended sessions. In all the analyses, we dichotomized the variables age (<60 years vs. ≥ 60 years) and number of attended sessions (<8 sessions vs. ≥ 8 sessions) to facilitate the interpretation of the model (see Supplementary Material). Model 3 was an exploratory linear mixed-effects model that accounted for repeated measures of the primary and secondary outcomes to investigate long-term effects. However, in the following, we do not interpret the statistical significance of Model 3 but rather descriptively explain the outcomes. For all the outcome variables, we created models with the interaction term of the variables condition and measurement time (t_1_ to t_3_) and the baseline values as fixed factors. Time, participant ID, and intercepts were treated as random factors to account for individual variability (see Supplementary Material). Model 4 included the same covariates as defined for Model 2 (see Supplementary Material).

Furthermore, we conducted a sensitivity analysis using Model 1 to examine heterogeneity in the intervention effect by animal species exposure (see Supplementary Material). Because the number of animal species was defined only within the AAT group and not applicable to the TAU group, species exposure could not be included as a covariate in the between-group models. Instead, heterogeneity in the AAT intervention was examined through an exploratory within-AAT analysis that assessed whether the number of animal species (exposure to one animal species vs. exposure to two or more animal species) was associated with socioemotional skills at post-assessment (t_1_), controlling for baseline levels.

Finally, we performed exploratory analyses of the primary outcomes to investigate the contribution of individual behavioral components at post-assessment (t_1_). Because social behavior and negative emotions were calculated as sum scores of several coded variables, we examined the association of each component variable with the treatment condition to better understand potential drivers of the observed effects. These analyses were conducted using Model 1 for each individual variable (see Supplementary Material).

## 3 Results

### 3.1 Participant characteristics

**Figure 5:**
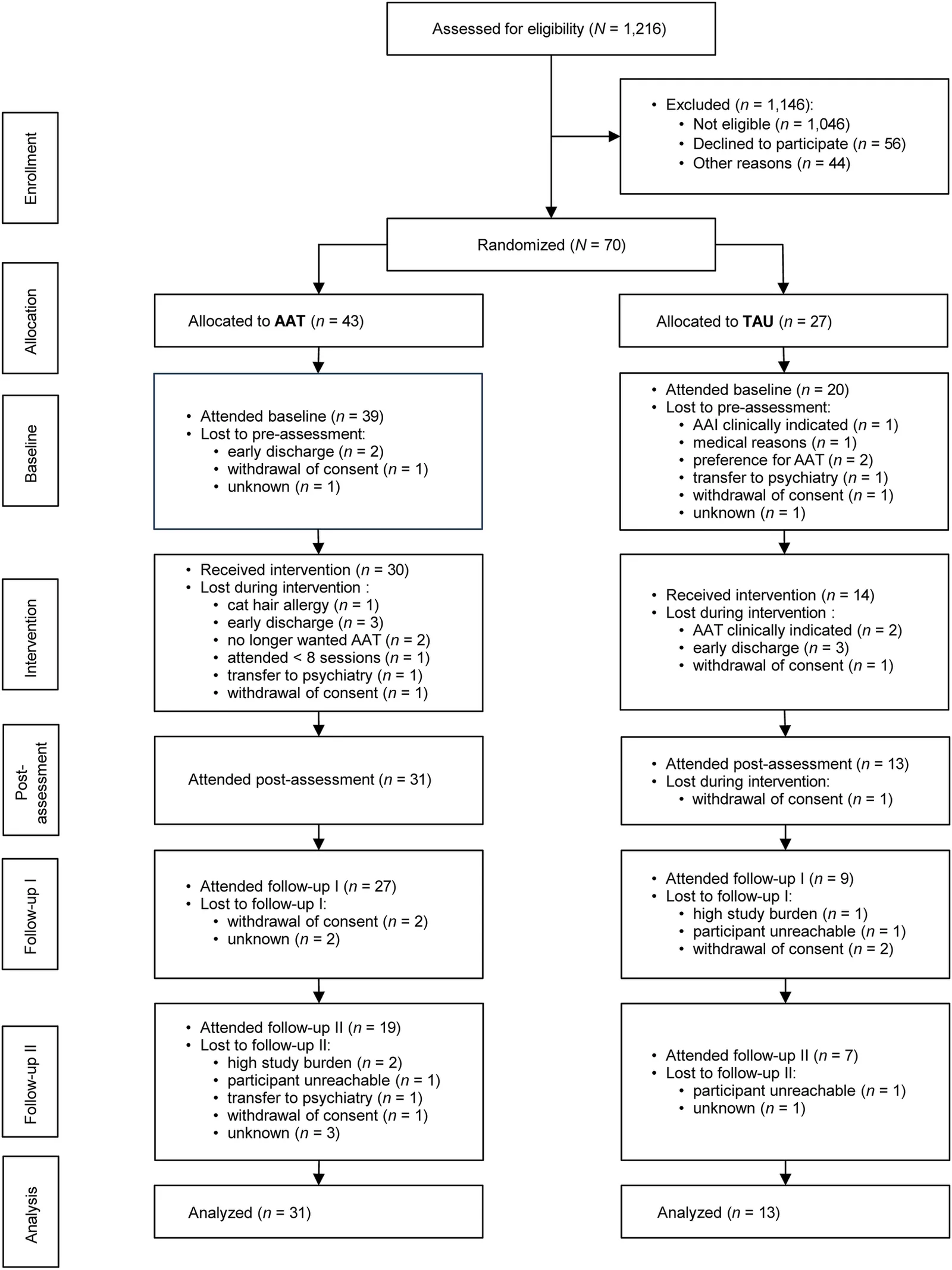
CONSORT flowchart of participant enrollment, allocation, follow-up, and analysis. The “Analysis” stage represents the mITT sample, comprising all randomized participants who completed at least one outcome assessment at post-assessment (t_1_). AAT, animal-assisted therapy; mITT, modified intention-to-treat; TAU, treatment as usual.

Between October 2018 and June 2024, 1,216 patients were assessed for eligibility, and 70 patients were randomized—43 to the AAT group and 27 to the TAU group. In the AAT group, 30 participants received the allocated intervention (*drop-out rate* = 30.23%), whereas 14 participants in the TAU group received the allocated intervention (*drop-out rate* = 48.15%). By the end of the study at follow-up II (t_3_), participant retention had decreased to 19 in the AAT group (*drop-out rate* = 55.81%) and 7 in the TAU group (*drop-out rate* = 74.07%) due to various reasons such as early discharge, withdrawal of informed consent, allergy, medical reasons, transfer to another institution, high study burden, did not want the allocated intervention, or unknown reasons (see Figure 5).

The total sample (*N* = 70) comprised 51 males (72.86%) and 19 (27.14%) females with an average age of 51.04 (*SD* = 13.39) years. Hemorrhage was the most common cause of ABI in both groups, followed by traumatic brain injury in the AAT group and other forms of nontraumatic brain injury in the TAU group. The time between the day of the injury (DOI) and the baseline assessment (t_0_) ranged from 18 to 1,318 days, and participants in the TAU group had, on average, a longer interval since the DOI (*M* = 179.41, *SD* = 286.41) than those in the AAT group (*M* = 123.35, *SD* = 66.24). At baseline, functional independence was moderately high for both groups (AAT: *M* = 89.73, *SD* = 16.90; TAU: *M* = 87.87, *SD* = 16.30). Session attendance averaged around 10 sessions per patient and mainly consisted of occupational therapy, followed by physiotherapy and speech therapy. Compared to the TAU group, patients in the AAT group attended fewer occupational therapy sessions (AAT: *M* = 5.62, *SD* = 3.95; TAU: *M* = 7.53, *SD* = 4.10) and more speech therapy sessions (AAT: *M* = 1.39, *SD* = 2.81; TAU: *M* = 0.80, *SD* = 2.83), while physiotherapy sessions were comparable between groups. The majority of the AAT group interacted with more than one animal species, guinea pigs and horses being the most common choice. Table 1 presents the demographic and clinical characteristics of all randomized participants (*N* = 70), as well as the intervention characteristics. Table 2 presents descriptive statistics for the primary and secondary outcomes across all assessment timepoints based on all available observations. Table 3 presents the primary and secondary analyses at post-assessment (t_1_) for the modified intention-to-treat sample (*N* = 44).

### 3.2 Primary outcomes: socioemotional skills at post-assessment

A total of 164 videos were analyzed to assess socioemotional skills, of which 106 (64.63%) were recorded during the COVID-19 pandemic. In most cases, researchers wore transparent face shields (*n* = 104, 63.41%), whereas patients wore face masks in only 2 videos (1.22%). At baseline (t_0_), the AAT and TAU groups showed similar levels of social behavior (AAT: *M* = 38.33, *SD* = 2.41; TAU: *M* = 38.98, *SD* = 2.82). The descriptive trends over time suggest that social behavior increased from baseline (t_0_) to post-assessment (t_1_) in the AAT group and remained relatively stable thereafter, whereas it showed little change over time in the TAU group (see Table 2). There were no baseline differences in social behavior between study completers and non-completers (see Supplementary Table 2). The primary between-group analysis at post-assessment (t_1_) (Model 1) showed that the patients in the AAT group displayed significantly more social behavior than those in the TAU group (*difference* = 3.24; 95% CI [0.87, 5.62]; *p* = .009). Baseline social behavior was also associated with social behavior at post-assessment (t_1_) (*difference* = 0.84; 95% CI [0.42, 1.26]; *p* < .001; see Table 3). The adjusted analysis, including the covariates (Model 2), did not show any additional statistically significant effects, suggesting that the covariates did not substantially alter the findings (see Supplementary Table 3). Exploratory longitudinal analyses evaluating the effects at follow-up (Models 3 and 4) are summarized in Supplementary Tables 4, 5. Sensitivity analyses showed no statistically significant association between animal species exposure and social behavior (see Supplementary Table 6). Exploratory *post hoc* analyses examining the contribution of individual behavioral components suggested that most of the variables (reactive vocalization, active questions, nondirected vocalization, body movement, eye contact, and prosocial behavior) were significantly associated with treatment condition (all *p* < .049). In contrast, active vocalization, baseline undefined vocalization, and baseline body movement were not significantly associated (see Supplementary Table 7).

Positive emotions showed largely stable trajectories over time, remaining unchanged between baseline (t_0_) and follow-up I (t_2_), with a slight increase between follow-up I (t_2_) and follow-up II (t_3_) in the AAT group. For the TAU group, positive emotions decreased after baseline (t_0_) and increased again between follow-up I (t_2_) and follow-up II (t_3_) (see Table 2). There were no statistically significant differences in baseline (t_0_) positive emotions between study completers and non-completers (see Supplementary Table 2). Model 1 revealed no statistically significant effect of condition on positive emotions (see Table 3). Model 2 indicated that age (*difference* = 7.41; 95% CI [0.36, 14.45]; *p* = .040) and expressed positive emotions of research staff (*difference* = 0.40; 95% CI [0.10, 0.70]; *p* = .011) were associated with expressed positive emotions in patients with ABI at post-assessment (t_1_) (see Supplementary Table 3). The results of Models 3 and 4, which explored the effects at follow-up, are summarized in the Supplementary Material (see Supplementary Tables 4, 5). Sensitivity analyses showed no statistically significant association between animal species exposure and positive emotions (see Supplementary Table 6).

Negative emotions were rarely observed: They occurred in less than 1% of the standardized interactions for both groups. No clear temporal trend or group differences in negative emotions were observable (see Table 2). There were no statistically significant differences in baseline (t_0_) negative emotions between study completers and non-completers (see Supplementary Table 2). Model 1 did not show a statistically significant effect of condition on negative emotions (see Table 3). Model 2 did not reveal any statistically significant associations between negative emotions displayed and the covariates during the standardized social interaction at post-assessment (t_1_) (see Supplementary Table 3). The results of Models 3 and 4, which explored the effects at follow-up, are summarized in the Supplementary Material (see Supplementary Tables 4, 5). Sensitivity analyses showed no statistically significant association between animal species exposure and negative emotions (see Supplementary Table 6). We conducted exploratory analyses to examine the influence of the individual variables of negative emotions (astonishment, sadness, anger, and fear). It showed that there might be an association with the condition on an individual level (see Supplementary Table 7).

### 3.3 Secondary outcomes: depressive-symptom severity, social cognition, therapy motivation, quality of life, and therapy adherence at post-assessment

Depressive symptom severity was low at baseline (BDI-FS < 3 in both groups) and showed a slight decrease over time in both groups (see Table 2). There were no statistically significant differences in baseline (t_0_) depressive symptom severity between study completers and non-completers (see Supplementary Table 2). Model 1 showed that the BDI-FS values at baseline (t_0_) were significantly associated with the BDI-FS values at post-assessment (t_1_) (*difference* = 0.32; 95% CI [0.03, 0.61]; *p* = .033). However, no statistically significant association between depressive symptom severity and condition could be detected (see Table 3). Model 2 showed no statistically significant effects of the covariates (see Supplementary Table 3). The results of Models 3 and 4, which explored the effects at follow-up, are summarized in the Supplementary Material (see Supplementary Tables 4, 5). Sensitivity analyses showed no statistically significant association between animal species exposure and depressive symptom severity (see Supplementary Table 6).

Social cognition increased over time in both groups in the exploratory longitudinal mixed-effects analyses (Model 3). However, both groups showed deficits in theory-of-mind abilities, with relatively low total ToM scores below 30 points (see Table 2). There were no statistically significant differences in baseline (t_0_) social cognition between study completers and non-completers (see Supplementary Table 2). Model 1 showed that the ToM scores at baseline (t_0_) were significantly associated with the ToM scores at post-assessment (t_1_) (*difference* = 0.77; 95% CI [0.56, 0.99]; *p* < .001). There was no statistically significant association between social cognition and condition (see Table 3). Model 2 did not reveal any statistically significant effects of the covariates (see Supplementary Table 3). The results of Models 3 and 4, explored the effect at follow-up, are summarized in the Supplementary Material (see Supplementary Tables 4, 5). Sensitivity analyses showed no association between animal species exposure and social cognition (see Supplementary Table 6).

Therapy motivation, which was assessed through the six FPTM subscales (psychological distress, hope for improvement, denial of the need for psychological help, knowledge about psychotherapy, initiative in seeking treatment, and symptom-related care by others), remained largely stable over time in the exploratory longitudinal mixed-effects analyses (Model 3). Among the subscales, hope showed the highest values, followed by symptom-related care (see Table 2). There were no statistically significant differences in baseline (t_0_) therapy motivation between study completers and non-completers (see Supplementary Table 2). Model 1 showed that the baseline (t_0_) values were significantly associated with the post-assessment (t_1_) values for psychological distress (*difference* = 0.67; 95% CI [0.42, 0.92]; *p* < .001), hope (*difference* = 0.49; 95% CI [0.23, 0.76]; *p* < .001), denial (*difference* = 0.64; 95% CI [0.36, 0.92]; *p* < .001), initiative (*difference* = 0.58; 95% CI [0.30, 0.86]; *p* < .001), and symptom-related care (*difference* = 0.40; 95% CI [0.15, 0.65]; *p* = .002). However, the condition did not have a statistically significant effect on any of the six subscales (see Table 3). Model 2 did not reveal any statistically significant effects of the covariates (see Supplementary Table 3). The results of Models 3 and 4, which explored the effects at follow-up, are summarized in Supplementary Tables 4, 5. Supplementary Table 6 shows that within the AAT group, exposure to a higher number of animal species was significantly associated with higher psychological distress scores (*estimate* = 0.61; 95% CI [0.11, 1.10], *p* = .018).

Quality of life, which was assessed through the four WHOQOL-BREF subscales (physical health, psychological well-being, social relationships, and environment), showed a slight increasing trend over time in the exploratory longitudinal mixed-effects analyses (Model 3). Overall quality of life appeared relatively high in this population, while physical health and psychological well-being showed the lowest scores across both groups (see Table 2). There were no statistically significant differences in baseline (t_0_) quality of life between study completers and non-completers (see Supplementary Table 2). Model 1 showed that the baseline (t_0_) values were significantly associated with the post-assessment (t_1_) values for physical health (*difference* = 0.48; 95% CI [0.26, 0.71]; *p* < .001), psychological well-being (*difference* = 0.46; 95% CI [0.28, 0.63]; *p* < .001), social relationships (*difference* = 0.65; 95% CI [0.38, 0.92]; *p* < .001), and environment (*difference* = 0.50; 95% CI [0.27, 0.74]; *p* < .001). The condition did not have a statistically significant effect on any of the four subscales of quality of life (see Table 3). Model 2 showed no statistically significant effect of the covariates at post-assessment (t_1_) (see Supplementary Table 3). The results of Models 3 and 4, which explored effects at follow-up, are summarized in the Supplementary Material (see Supplementary Tables 4, 5). Supplementary Table 6 shows that within the AAT group, exposure to two or more animal species was significantly associated with lower scores for environmental quality of life compared with exposure to one species (*estimate* = −13.3; 95% CI [−24.0, −2.50], *p* = .018).

Therapy adherence among participants who completed the post-assessment (t_1_) was relatively high, with an average of *M* = 9.71 (*SD* = 3.28, 80.88%) attended sessions in the AAT group and an average of *M* = 10.47 (*SD* = 2.85, 87.23%) attended sessions in the TAU group out of the 12 planned sessions (see Table 1). The unadjusted and adjusted models both show a strong and statistically significant association between completion status and therapy adherence. Specifically, study completers attended a substantially higher proportion of therapy sessions than non-completers (*unadjusted estimate* = 0.68; 95% CI [0.45, 0.91], *p* < .001; *adjusted estimate* = 0.69; 95% CI [0.46, 0.92], *p* < .001). Model 1 did not show any statistically significant effect of the condition on therapy adherence (see Table 3), and Model 2 also did not reveal any statistically significant effects of the covariates (see Supplementary Table 3). The results of Models 3 and 4, which explored the effects at follow-up, are summarized in Supplementary Tables 4, 5. Sensitivity analyses showed no statistically significant association between exposure to animal species and therapy adherence (see Supplementary Table 6).

## 4 Discussion

This study explored whether AAT for adult patients with ABI in inpatient neurorehabilitation was associated with improvements in socioemotional skills beyond therapy sessions, particularly in social interactions. Compared to patients in the TAU group, patients who received 6 weeks of AAT showed significantly more social behavior at post-assessment (t_1_) during a standardized social interaction outside the therapy context designed to approximate real-life situations. We did not find any statistically significant group effects on positive and negative emotions, depressive symptom severity, social cognition, therapy motivation, quality of life, or adherence to treatment. However, as the preregistered primary endpoint at 18 weeks (t_3_) could not be meaningfully analyzed due to substantial attrition, the results at post-assessment (t_1_) should be interpreted as exploratory rather than confirmatory. Exploratory longitudinal mixed-effects analyses further indicated largely stable trajectories of socioemotional outcomes over time in both groups, with only a modest increase in social behavior from baseline to post-assessment in the AAT group and no clear long-term group-specific patterns. These findings must be interpreted cautiously given the limited number of observations at later time points due to high attrition.

### 4.1 AAT and socioemotional skills beyond the therapy context

The finding that patients showed more social behavior after being treated with AAT aligns with previous reports. In a previous controlled study, patients with ABI showed increased social competence and higher social engagement during therapies in the presence of an animal compared to similar therapies without the presence of an animal (44). Another study demonstrated that animals serve as social catalysts that encourage children with severe neurological impairments to engage more with others and to adhere to group norms (47). Research on dog-assisted therapy has suggested that the nonjudgmental presence of animals particularly supports patients with communication impairments, such as aphasia, facilitating more active engagement in therapy sessions (79). Also, animals were found to be important social partners who provide warmth and protection, effectively reducing feelings of loneliness, enhancing the social support system and meeting socioemotional needs for individuals during rehabilitation (45, 47).

Exploratory analyses at post-assessment (t_1_) did not indicate any statistically significant group differences for other socioemotional outcomes, including positive and negative emotions, depressive symptom severity, social cognition, therapy motivation, quality of life, and therapy adherence. This contrasts with previous studies that have reported improvements in positive emotions, in-session motivation, quality of life, and therapy adherence, reduced distress and depressive symptoms for people with ABI receiving AAT (17, 42, 44, 45). One possible reason for this could be that the patients who agreed to join our study already exhibited low levels of depressive symptoms, strong motivation for therapy, and a high quality of life at the start of the study. This suggests a potential ceiling effect, where participants already possessed relatively high socioemotional skills that could not be significantly enhanced over time (80). In addition, evidence indicates that individuals with acquired brain injuries tend to benefit most from early-onset neurorehabilitation, with the greatest gains in functioning typically observed during the acute phase of recovery (81). Because our study was conducted in the post-acute phase, the intervention may have occurred at a time when treatment progress had already begun to plateau (82). More severely affected patients or those in earlier stages of rehabilitation might have demonstrated different progressions over time. Another possible explanation for why we did not find any effects of AAT on depressive-symptom severity, therapy motivation, and quality of life could be the high drop-out rate, which diminished the statistical power and the likelihood of detecting any effects. Furthermore, additional analyses indicated that individual- and intervention-related factors may have influenced the outcomes. Covariate analyses showed an association between age and negative emotions. Exploratory within-group analyses further suggested that exposure to a higher number of animal species within the AAT group was associated with higher psychological distress and lower environmental quality of life. These findings should be interpreted cautiously, given their exploratory nature and may reflect contextual factors or limited opportunities for forming stable therapeutic bonds rather than effects of species diversity itself. The effects might also have been larger in a sample of patients who were more motivated for AAT. We noticed that many patients declined to participate due to the risk of being assigned to the control group without AAT, so some highly motivated patients who wanted AAT did not participate. Although we did not find any effect of AAT on therapy adherence, there was a clear group difference regarding drop-out rate, with a higher drop-out rate of 74.07% in the control group compared to 55.81% in the AAT group by the end of the study. While the drop-out rate was lower in the AAT group, it remained above 50%, which is not in line with a previous study on AAT in neurorehabilitation, which reported high treatment adherence rates, suggesting strong patient acceptance among patients with ABI (45). To evaluate the risk of attrition bias, we compared baseline characteristics of study completers and non-completers and found no statistically significant differences in socioemotional, psychological, or cognitive outcomes, suggesting that attrition was not systematically associated with baseline outcome severity. Differences in therapy adherence between completers and non-completers were expected by definition and reflect study completion status rather than meaningful baseline imbalance. Most reasons for discontinuation were of organizational nature. Loss to follow-up is common in longitudinal studies of patients with ABI, and higher attrition has been linked to greater follow-up frequency, single-center designs, mild ABI, and noncontact outcome collection (83). In our case, reducing the burden of follow-up visits with telephone or virtual study appointments was not feasible due to the nature of the standardized social interaction.

### 4.2 Strengths and limitations

To our knowledge, this study is the first to explore whether the effects of AAT on socioemotional skills transfer from the therapy context to daily-life social interactions in adult patients with ABI undergoing inpatient rehabilitation. The randomized controlled design with repeated measurements provided a structured framework to explore between-group and within-group changes over time. Our study also benefited from a diverse patient population that included individuals with traumatic and non-traumatic brain injuries, making our findings more broadly applicable to various rehabilitation settings where such distinctions often do not dictate treatment paths. Furthermore, including an active control condition and the standardization of therapy sessions enhanced the reliability of our interventions and improved the comparability between the groups. We utilized a combination of behavior analyses, tests, and self-reports to measure outcomes, effectively reducing detection biases and increasing the accuracy of our results. This is crucial for patients with cognitive impairments, which are typical after ABI. The interdisciplinary approach of our study incorporated experienced therapists from occupational therapy, speech therapy, and physiotherapy, as well as trained animals and animal handlers, ensuring a safe and ethical treatment of the animals aligned with the One Health approach and IAHAIO guidelines (21). By integrating various therapy modalities and animal species, our study reflected a real-world rehabilitation scenario, increasing its external validity. Finally, the use of a modified intention-to-treat approach allowed the inclusion of all available post-assessment data, maximizing transparency and data use despite substantial attrition.

However, our study has several limitations. The absence of blinding introduced potential performance bias in patients as well as raters of the videos, as they were aware of group allocation after the first session. Despite our efforts to mitigate this through a rigorous coding scheme and ensure high interrater reliability, bias cannot be fully excluded. The small sample size reduced the statistical power and increased the risk of type II errors. The results thus need to be interpreted carefully.

The high drop-out rates raise concerns about attrition bias. Many patients found the follow-up assessments burdensome after discharge from the clinic. Other challenges arose from participants’ packed schedules and the perceived burden during the study for both patients and staff. Due to substantial attrition, the preregistered primary endpoints at follow-up II (t_3_) could not be analyzed, constituting a protocol deviation and limiting analyses to an exploratory examination of post-assessment (t_1_) outcomes. Furthermore, many patients were unwilling to risk not receiving AAT. This led to recruitment difficulties and a potential selection bias toward participants who were less motivated for AAT. Additionally, patients who agreed to participate in the study were generally high functioning and reported low depressive symptoms, high therapy motivation, and a high quality of life, which limits the generalizability of the study to the broader population of patients with ABI. Although no significant baseline differences were found between completers and non-completers, attrition bias cannot be ruled out, given the small sample size. Moreover, retention may have been influenced by both participant characteristics and rehabilitation-related factors. Patients who were more socially engaged, motivated, or clinically stable may have been more likely to remain in the study and complete follow-up assessments. Conversely, patients with greater social difficulties, lower motivation, or earlier discharge may have been underrepresented among completers. At the same time, patients with more favorable rehabilitation trajectories may have been discharged earlier and therefore become less available for follow-up assessments. Such selective retention may have affected the interpretation of the findings, as participants who completed the study may not have been fully representative of the broader ABI population. Depending on which patient groups were preferentially retained, the observed treatment effects may have been either over- or underestimated. While therapy motivation and aspects of clinical severity were assessed at baseline, not all relevant factors (e.g., discharge timing, discharge destination, and rehabilitation progression) were captured or fully accounted for in the attrition analyses. Therefore, the magnitude and direction of any attrition-related bias remain uncertain.

The randomization procedure represents a further limitation. It was based on a simple Excel-generated sequence without allocation constraints or stratification, resulting in imbalances in group size, age, and gender (1.61:1 ratio favoring AAT). These differences may have affected the responsiveness to treatment and introduced confounding effects. For instance, age-related factors may affect emotional regulation and engagement in therapy (84). In addition, the timing, number, and type of rehabilitation sessions varied between groups, with the AAT group receiving relatively more speech therapy and less occupational therapy. As speech therapy specifically targets social communication, this imbalance may have influenced socioemotional outcomes (85, 86). However, given the low number of speech therapy sessions and the multidisciplinary rehabilitation context (87), it is unlikely to fully explain the observed effects. More broadly, socioemotional functioning during inpatient neurorehabilitation is influenced by multiple contextual factors, including rehabilitation progression, interactions with staff and relatives, and differences in the overall therapeutic environment. Consequently, the observed group differences cannot be attributed exclusively to AAT with certainty. Future studies employing block or stratified randomization and more standardized therapy exposure may help reduce these potential sources of bias.

Limitations in measurement and generalizability should also be acknowledged. The primary outcome reflected performance within a structured observational paradigm designed to approximate real-life social interactions rather than direct assessment of social functioning in everyday life. Our standardized social situation has not been validated in ABI populations, and the behavioral paradigm, including the composite score and its weighting, remains clinically and theoretically informed rather than formally validated as a measure of broader social participation. Consequently, while the findings indicate changes in observed social behavior within the assessment setting, the extent to which these effects generalize to everyday social interactions remains uncertain. Furthermore, although the MASC captures complex and ecologically relevant aspects of social cognition, it has not been specifically validated in ABI populations, which may limit the interpretation of social-cognition findings in this context. In addition, a single-center study limits the generalizability across diverse clinical, cultural contexts, although this was optimized for internal validity.

Finally, contextual factors related to the extended data collection period should be considered. Data collection occurred over an extended period (November 2018 to June 2024), with 64.63% of participants assessed during the COVID-19 pandemic, during which either patients, researchers, or both wore masks or transparent face shields. These conditions likely impacted social behavior (e.g., reduced facial expressivity, altered eye contact, reduced face-to-face interaction, etc.), and introduced challenges for behavioral-coding, potentially affecting measurement reliability (88). Beyond these measurement-related issues, the COVID-19 pandemic substantially altered rehabilitation environments and opportunities for social participation (89–91). Infection-control measures often resulted in visitor restrictions, reduced group activities, periods of isolation or quarantine, and fewer opportunities for informal social interaction within rehabilitation settings (92–94). Reduced social contact and increased loneliness, stress, and uncertainty may therefore have affected socioemotional functioning independently of the intervention and influenced how patients expressed social behavior during the standardized social situation (95–97). Consequently, observed social behavior may partly reflect the broader social context in which participants were assessed rather than solely their underlying socioemotional abilities. At the same time, participation in the study may itself have provided opportunities for structured social interaction during a period when many usual social contacts were restricted. This may have been particularly relevant for the AAT group, as interactions with animals can provide companionship, emotional support, and additional opportunities for engagement (30, 32, 36), potentially contributing to differential study retention between groups. Furthermore, pandemic-related uncertainty and concerns about infection risk may have influenced recruitment by discouraging participation among more socially isolated or vulnerable patients, who may have exhibited different socioemotional trajectories or responded differently to AAT, thereby limiting the generalizability of the findings (94–96).

### 4.3 Future research and practical implications

Our finding that AAT can lead to improved functioning in a social situation outside therapy is clinically highly relevant. Future research should dive more into this potential of AAT by systematically addressing the longer-term effects of AAT, its transferability to patients’ daily lives, and its possible effects on longer-term rehabilitation outcomes. Assessing the long-term effects of AAT beyond inpatient neurorehabilitation could provide insights into the therapy's impact on daily life (44). Future studies should consider a larger, more-balanced sample size and adopt a multicenter approach to enhance generalizability. Assessing rehabilitation goals with independent, standardized tools and including perspectives from patients, relatives, and therapists through quantitative and qualitative assessments could provide a more holistic context for the findings. Whether the observed effects on social behavior within the standardized observational paradigm translate to everyday social interactions should be addressed using more naturalistic and ecologically valid outcome measures. Additionally, incorporating assessments of premorbid socioemotional skills may be important, as they potentially correlate with treatment outcomes (16). The low eligibility rate and high attrition observed in this study primarily reflect the challenges of conducting randomized longitudinal trials in patients with ABI rather than the limited feasibility of AAT itself. Cognitive impairments, fatigue, and the burden of repeated assessments may restrict trial participation and retention, and selection bias may arise if patients who are less motivated for AAT are more likely to enroll. Future studies could reduce follow-up burden by conducting home-based assessments, integrating standardized interaction tasks into routine clinical appointments, or shortening assessment protocols, thereby maintaining ecological validity while minimizing participant burden. Hybrid approaches, such as the remote administration of questionnaires combined with in-person interaction tasks or the cautious adaptation of standardized social interaction paradigms to virtual formats, may further enhance feasibility, although their validity for assessing real-world social behavior requires careful evaluation. Moreover, the lack of blinding and the organizational demands placed on clinical staff further complicate the implementation of standard randomized controlled trials in this setting. Given that AAT is already routinely implemented in neurorehabilitation (20, 24, 25), future research should consider alternative, more pragmatic and patient-centered study designs (e.g., multiperiod crossover trials, pragmatic trials, or multiple-case-study designs) to better evaluate the feasibility and effectiveness of AAT in real-world neurorehabilitation contexts (98, 99).

### 4.4 Conclusion

In conclusion, due to the inability to analyze the preregistered primary endpoint at 12-week follow-up and the resulting reliance on exploratory post-assessment analyses, this study provides exploratory findings suggesting that AAT may enhance social behavior in a standardized social situation, indicating potential transfer effects beyond the immediate therapy context of inpatient neurorehabilitation. As socioemotional functioning is essential for the physical and psychological well-being of patients with ABI, these findings indicate that AAT may represent a promising complementary approach for improving socioemotional skills in neurorehabilitation. However, given the exploratory nature of the analyses, the absence of blinding, high drop-out rates, and the limited sample size, the results should be interpreted with caution. Future research should replicate these findings in larger and more diverse samples, ensure adherence to preregistered primary endpoints, and investigate the long-term and real-world impact of AAT on broader aspects of psychosocial functioning and rehabilitation outcomes. Moreover, evaluating patient preferences and alternative designs suited to complex clinical contexts may help refine and optimize the use of AAT in inpatient neurorehabilitation. Clarifying its specific benefits, mechanisms, and optimal conditions of use is essential to effectively integrating AAT into evidence-based rehabilitation programs.

## Data Availability

The datasets presented in this study can be found in online repositories. The name of the repository and accession number can be found here: https://doi.org/10.17605/OSF.IO/8JXYH.

## References

[1] Maas AIR Menon DK Adelson PD Andelic N Bell MJ Belli A. Traumatic brain injury: integrated approaches to improve prevention, clinical care, and research. Lancet Neurol. (2017) 16:987–1048. 10.1016/S1474-4422(17)30371-X 29122524

[2] Kusec A Panday J Froese A Albright H Harris JE. Getting motivated: long-term perspectives on engaging in community-based programs after acquired brain injury. Brain Inj. (2020) 34:1331–8. 10.1080/02699052.2020.1802657 32780592

[3] Last J Perrech M Denizci C Dorn F Kessler J Seibl-Leven M. Long-term functional recovery and quality of life after surgical treatment of putaminal hemorrhages. J Stroke Cerebrovasc Dis. (2015) 24:925–9. 10.1016/j.jstrokecerebrovasdis.2014.12.001 25804566

[4] Azouvi P Arnould A Dromer E Vallat-Azouvi C. Neuropsychology of traumatic brain injury: an expert overview. Rev Neurol (Paris). (2017) 173:461–72. 10.1016/J.NEUROL.2017.07.006 28847474

[5] Camilotto N Caamaño P Leiva S. Behavioral changes after traumatic brain injury: a systematic review and meta-analysis on the frontal systems behavior scale. Int J Psychol Res (Medellin). (2024) 17:112–29. 10.21500/20112084.6414

[6] Kusec A Velikonja D Dematteo C Harris JE. Motivation in rehabilitation and acquired brain injury: can theory help us understand it? Disabil Rehabil. (2019) 41:2343–9. 10.1080/09638288.2018.1467504 29693464

[7] Ryan NP Catroppa C Godfrey C Noble-Haeusslein LJ Shultz SR O’Brien TJ. Social dysfunction after pediatric traumatic brain injury: a translational perspective. Neurosci Biobehav Rev. (2016) 64:196–214. 10.1016/J.NEUBIOREV.2016.02.020 26949224 PMC5627971

[8] Milders M. Relationship between social cognition and social behaviour following traumatic brain injury. Brain Inj. (2019) 33:62–8. 10.1080/02699052.2018.1531301 30325217

[9] Dethier M Blairy S Rosenberg H McDonald S. Spontaneous and posed emotional facial expressions following severe traumatic brain injury. J Clin Exp Neuropsychol. (2012) 34:936–47. 10.1080/13803395.2012.702734 22827155

[10] MacDonald S. Introducing the model of cognitive-communication competence: a model to guide evidence-based communication interventions after brain injury. Brain Inj. (2017) 31:1760–80. 10.1080/02699052.2017.1379613 29064304

[11] Cassel A McDonald S Kelly M Togher L. Learning from the minds of others: a review of social cognition treatments and their relevance to traumatic brain injury. Neuropsychol Rehabil. (2019) 29:22–55. 10.1080/09602011.2016.1257435 27899036

[12] Wiseman-Hakes C Ryu H Lightfoot D Kukreja G Colantonio A Matheson FI. Examining the efficacy of communication partner training for improving communication interactions and outcomes for individuals with traumatic brain injury: a systematic review. Arch Rehabil Res Clin Transl. (2020) 2:100036. 10.1016/J.ARRCT.2019.100036 33543065 PMC7853340

[13] Maggio MG Maresca G Stagnitti MC Anchesi S Casella C Pajno V. Social cognition in patients with acquired brain lesions: an overview on an under-reported problem. Appl Neuropsychol Adult. (2022) 29:419–31. 10.1080/23279095.2020.1753058 32301351

[14] Grewal J Citton K Sing G Biagioni JB Schmidt J. Priorities for quality of life after traumatic brain injury. PLoS One. (2024) 19:e0306524. 10.1371/JOURNAL.PONE.0306524 38968208 PMC11226113

[15] Karunanithi G Sagar RP Joy A Vedasoundaram P. Assessment of psychological distress and its effect on quality of life and social functioning in cancer patients. Indian J Palliat Care. (2018) 24:72–7. 10.4103/IJPC.IJPC_104_17 29440811 PMC5801634

[16] Hart T Brenner L Clark AN Bogner JA Novack TA Chervoneva I. Major and minor depression after traumatic brain injury. Arch Phys Med Rehabil. (2011) 92:1211–9. 10.1016/j.apmr.2011.03.005 21807140

[17] An HJ Park SJ. Effects of animal-assisted therapy on gait performance, respiratory function, and psychological variables in patients post-stroke. Int J Environ Res Public Health. (2021) 18:5818. 10.3390/ijerph18115818 34071529 PMC8198745

[18] Friedler B Crapser J McCullough L. One is the deadliest number: the detrimental effects of social isolation on cerebrovascular diseases and cognition. Acta Neuropathol. (2015) 129:493–509. 10.1007/S00401-014-1377-9 25537401 PMC4369164

[19] Antonucci SM. Animal-assisted intervention in speech-language pathology: practical, clinical, and theoretical considerations. Semin Speech Lang. (2022) 43:1–7. 10.1055/S-0041-1741555 35135018 PMC10108850

[20] Mittly V Farkas-Kirov C Zana Á Szabó K Ónodi-Szabó V Purebl G. The effect of animal-assisted interventions on the course of neurological diseases: a systematic review. Syst Rev. (2023) 12:224. 10.1186/S13643-023-02387-Y 38007472 PMC10675848

[21] IAHAIO. IAHAIO White Paper 2014, updated for 2018. The IAHAIO definitions for animal assisted intervention and guidelines for wellness of animals involved in AAI (2018). Available online at: https://iahaio.org/wp/wp-content/uploads/2021/01/iahaio-white-paper-2018-english.pdf (Accessed April 25, 2025)

[22] Kovács KE Balogh ÉZ Lovas B Boris P Nagy BE. The role of animal-assisted programs in physical health improvement of children and adolescents with special education needs - a systematic review. BMC Public Health. (2024) 24:824. 10.1186/S12889-024-18326-Y PMC10943833 38491498

[23] Wood WH Fields BE. Hippotherapy: a systematic mapping review of peer-reviewed research, 1980 to 2018. Disabil Rehabil. (2021) 43:1463–87. 10.1080/09638288.2019.1653997 31491353

[24] Lasa SM Bocanegra NM Alcaide RV Arratibel MAA Donoso EV Ferriero G. Animal assisted interventions in neurorehabilitation: a review of the most recent literature. Neurologia. (2015) 30:1–7. 10.1016/J.NRL.2013.01.012 23642347

[25] Boldig CM Butala N. Pet therapy as a nonpharmacological treatment option for neurological disorders: a review of the literature. Cureus. (2021) 13:e16167. 10.7759/CUREUS.16167 34367777 PMC8336327

[26] Giannou I Katsina M Dimitriadis Z Paras G Besios T. The effect of hippotherapy on people with multiple sclerosis, a systematic review. Mult Scler Relat Disord. (2025) 97:106374. 10.1016/J.MSARD.2025.106374 40088721

[27] Wagner C Gaab J Hediger K. The importance of the treatment rationale for pain in animal-assisted interventions: a randomized controlled trial in healthy participants. J Pain. (2023) 24:1080–93. 10.1016/J.JPAIN.2023.01.004 36641027

[28] Serpell JA. Companion animals. In: Hosey G Melfi V, editors. Anthrozoology: Human-Animal Interactions in Domesticated and Wild Animals. Oxford: Oxford University Press. (2018). p. 17–31. 10.1093/OSO/9780198753629.003.0002

[29] Prato-Previde E Basso Ricci E Colombo ES. The complexity of the human-animal bond: empathy, attachment and anthropomorphism in human-animal relationships and animal hoarding. Animals (Basel). (2022) 12:2835. 10.3390/ANI12202835 36290219 PMC9597799

[30] Lu Y. A theoretical review of human-pet relationships from the perspective of attachment theory: toward interdisciplinary integration and conceptual reconstruction. Adv Soc Behav Res. (2025) 16:116–20. 10.54254/2753-7102/2025.25477

[31] Eckstein M Mamaev I Ditzen B Sailer U. Calming effects of touch in human, animal, and robotic interaction—scientific state-of-the-art and technical advances. Front Psychiatry. (2020) 11:555058. 10.3389/FPSYT.2020.555058 33329093 PMC7672023

[32] Northrope K Shnookal J Ruby MB Howell TJ. The relationship between attachment to pets and mental health and wellbeing: a systematic review. Animals (Basel). (2025) 15:1143. 10.3390/ani15081143 40281976 PMC12023967

[33] Uvnäs-Moberg K Gross MM Calleja-Agius J Turner JD Vaudry H Sona C. The yin and yang of the oxytocin and stress systems: opposites, yet interdependent and intertwined determinants of lifelong health trajectories. Front Endocrinol (Lausanne). (2024) 15:1272270. 10.3389/FENDO.2024.1272270 PMC11058227 38689729

[34] Beetz AM. Theories and possible processes of action in animal assisted interventions. Appl Dev Sci. (2017) 21:139–49. 10.1080/10888691.2016.1262263

[35] McConnell AR Folmsbee JR Aldstadt KM. Furry friends: direct and indirect benefits of human-animal relationships on well-being. Soc Personal Psychol Compass. (2025) 19:e70091. 10.1111/SPC3.70091

[36] Wood L Martin K Christian H Nathan A Lauritsen C Houghton S. The pet factor - companion animals as a conduit for getting to know people, friendship formation and social support. PLoS One. (2015) 10:e0122085. 10.1371/journal.pone.0122085 25924013 PMC4414420

[37] McSween MP Day T Hill J Wallace SJ. Animal-assisted services for adults with acquired neurogenic communication disorders: a scoping review. Int J Lang Commun Disord. (2024) 59:2858–77. 10.1111/1460-6984.13119 39361024

[38] Cerulli C Murri A Grazioli E Tranchita E Tinè F De Santis Del Tavano C. Can animal assisted interventions counteract apathy and improve physical activity levels in psychiatric patients with cognitive disability? A case study. J Bodyw Mov Ther. (2024) 40:513–9. 10.1016/J.JBMT.2024.05.007 39593635

[39] Gocheva V Hund-Georgiadis M Hediger K. Effects of animal-assisted therapy on concentration and attention span in patients with acquired brain injury: a randomized controlled trial. Neuropsychology. (2018) 32:54–64. 10.1037/neu0000398 29035068

[40] Arnskötter W Marcar VL Wolf M Hund-Georgiadis M Hediger K. Animal presence modulates frontal brain activity of patients in a minimally conscious state: a pilot study. Neuropsychol Rehabil. (2022) 32:1324–36. 10.1080/09602011.2021.1886119 33602057

[41] Marti R Petignat M Marcar VL Hattendorf J Wolf M Hund-Georgiadis M. Effects of contact with a dog on prefrontal brain activity: a controlled trial. PLoS One. (2022) 17:e0274833. 10.1371/JOURNAL.PONE.0274833 36197880 PMC9534402

[42] Theis F Luck F Hund-Georgiadis M Hediger K. Influences of animal-assisted therapy on episodic memory in patients with acquired brain injuries. Int J Environ Res Public Health. (2020) 17:8466. 10.3390/ijerph17228466 33207575 PMC7696148

[43] Hediger K Petignat M Marti R Hund-Georgiadis M. Animal-assisted therapy for patients in a minimally conscious state: a randomized two treatment multi-period crossover trial. PLoS One. (2019) 14:e0222846. 10.1371/journal.pone.0222846 31574106 PMC6772068

[44] Hediger K Thommen S Wagner C Gaab J Hund-Georgiadis M. Effects of animal-assisted therapy on social behaviour in patients with acquired brain injury: a randomised controlled trial. Sci Rep. (2019) 9:5831. 10.1038/s41598-019-42280-0 30967589 PMC6456498

[45] Künzi P Ackert M Grosse Holtforth M Hund-Georgiadis M Hediger K. Effects of animal-assisted psychotherapy incorporating mindfulness and self-compassion in neurorehabilitation: a randomized controlled feasibility trial. Sci Rep. (2022) 12:10898. 10.1038/s41598-022-14584-1 PMC9240064 35764668

[46] Hediger K Boek F Sachers J Blankenburg U Antonius-Kluger E Rist B. Dog-assisted therapy in neurorehabilitation of children with severe neurological impairment: an explorative study. Neuropediatrics. (2020) 51:267–74. 10.1055/s-0040-1708545 32176927

[47] Hediger K Lunzenfichter M Markzoll E Arnskötter W Schaudek M Kluger G. Psychological aspects of hippotherapy for children with severe neurological impairment: an exploratory study. PLoS One. (2025) 20:e0320238. 10.1371/JOURNAL.PONE.0320238 40198572 PMC11978075

[48] Rodríguez-Martínez MDC De la Plana Maestre A Armenta-Peinado JA Barbancho MÁ García-Casares N. Evidence of animal-assisted therapy in neurological diseases in adults: a systematic review. Int J Environ Res Public Health. (2021) 18:12882. 10.3390/IJERPH182412882 PMC8701659 34948491

[49] O'Laughlin M Edwards R Bould E Devine S Downing S. Animal-assisted interventions in adult hospital rehabilitation settings: a scoping review. Nurs Health Sci. (2024) 26:e13138. 10.1111/NHS.13138 39013555

[50] Wagner C Grob C Hediger K. Specific and non-specific factors of animal-assisted interventions considered in research: a systematic review. Front Psychol. (2022) 13:931347. 10.3389/FPSYG.2022.931347 35837630 PMC9274084

[51] Menna LF Santaniello A Todisco M Amato A Borrelli L Scandurra C. The human–animal relationship as the focus of animal-assisted interventions: a one health approach. Int J Environ Res Public Health. (2019) 16:3660. 10.3390/IJERPH16193660 31569460 PMC6801464

[52] Beck AT Steer RA Brown GK. BDI-Fast Screen for Medical Patients: Manual. German adaptation by Kliem S, Brähler E. Frankfurt am Main: Pearson Assessment & Information. (2013).

[53] Furlanetto LM Mendlowicz MV Romildo Bueno J. The validity of the Beck depression inventory-short form as a screening and diagnostic instrument for moderate and severe depression in medical inpatients. J Affect Disord. (2005) 86:87–91. 10.1016/j.jad.2004.12.011 15820275

[54] Poole H Bramwell R Murphy P. The utility of the Beck Depression Inventory Fast Screen (BDI-FS) in a pain clinic population. Eur J Pain. (2009) 13:865–9. 10.1016/j.ejpain.2008.09.017 19010075

[55] Kliem S Mößle T Zenger M Brähler E. Reliability and validity of the Beck Depression Inventory–Fast Screen for medical patients in the general German population. J Affect Disord. (2014) 156:236–9. 10.1016/j.jad.2013.11.024 24480380

[56] Dziobek I Fleck S Kalbe E Rogers K Hassenstab J Brand M. Introducing MASC: a movie for the assessment of social cognition. J Autism Dev Disord. (2006) 36:623–36. 10.1007/s10803-006-0107-0 16755332

[57] Montag C Dziobek I Richter IS Neuhaus K Lehmann A Sylla R. Different aspects of theory of mind in paranoid schizophrenia: evidence from a video-based assessment. Psychiatry Res. (2011) 186:203–9. 10.1016/j.psychres.2010.09.006 20947175

[58] Lahera G Boada L Pousa E Mirapeix I Morón-Nozaleda G Marinas L. Movie for the assessment of social cognition (MASC): Spanish validation. J Autism Dev Disord. (2014) 44:1886–96. 10.1007/s10803-014-2061-6 24522969

[59] Ritter K Dziobek I Preißler S Rüter A Vater A Fydrich T. Lack of empathy in patients with narcissistic personality disorder. Psychiatry Res. (2011) 187:241–7. 10.1016/j.psychres.2010.09.013 21055831

[60] Chang YH Yu CL Huang CC Wang TY Dziobek I Lane HY. Discrepancy of social cognition between bipolar disorders and major depressive disorders. Brain Behav. (2024) 14:e3365. 10.1002/brb3.3365 38376012 PMC10757902

[61] Schulz H Nübling R Rüddel H. Entwicklung einer Kurzform eines Fragebogens zur Psychotherapiemotivation. Verhaltenstherapie. (1995) 5:89–95. 10.1159/000258901

[62] Gerke L Meyrose AK Ladwig I Rief W Nestoriuc Y. Frequencies and predictors of negative effects in routine inpatient and outpatient psychotherapy: two observational studies. Front Psychol. (2020) 11:2144. 10.3389/fpsyg.2020.02144 32982878 PMC7478145

[63] Nübling R Schulz H Schmidt J Koch U Wittmann WW. Fragebogen zur Psychotherapiemotivation (FPTM) – Testkonstruktion und Gütekriterien. In: Nübling R Muthny F Bengel J, editors. Reha-Motivation und Behandlungserwartung. Bern: Huber. (2006). p. 252–270.

[64] Scheidegger A Gómez Penedo JM Blättler LT Aybek S Bischoff N Grosse Holtforth M. How treatment motivation predicts favorable outcomes in interdisciplinary multimodal pain treatment among patients with chronic primary pain. J Clin Psychol Med Settings. (2024) 31:48–57. 10.1007/s10880-023-09958-0 37081250 PMC10924698

[65] Conrad I Matschinger H Kilian R Riedel-Heller SG. WHOQOL-OLD und WHOQOL-BREF: Handbuch für die deutschsprachigen Versionen der WHO-Instrumente zur Erfassung der Lebensqualität im Alter. Göttingen: Hogrefe. (2016).

[66] World Health Organization. WHOQOL user manual. WHO/HIS/HSI Rev.2012.03. Geneva: World Health Organization. (2012).

[67] Kasaudhan S Chaudhary V Saraswathy KN Dhamija RK Mahesh KSSU Devi NK. Impact of hypertension, diabetes, and obesity on quality of life in rural Punjab, India. Discover Public Health. (2024) 21:242. 10.1186/s12982-024-00359-8

[68] Abraha I Montedori A. Modified intention to treat reporting in randomised controlled trials: systematic review. BMJ. (2010) 340:c2697. 10.1136/BMJ.C2697 20547685 PMC2885592

[69] R Core Team. R: A Language and Environment for Statistical Computing. Vienna: R Foundation for Statistical Computing. (2025). Available online at: https://cran.r-project.org (Accessed February 5, 2026).

[70] Robinson D Hayes A Couch S Hvitfeldt E. Broom: Convert Statistical Objects into Tidy Tibbles [R package version 1.0.12]. CRAN: Contributed Packages. (2026). 10.32614/CRAN.PACKAGE.BROOM

[71] Bolker B Robinson D. Broom.mixed: Tidying Methods for Mixed Models [R package version 0.2.9.6]. CRAN: Contributed Packages. (2024). 10.32614/CRAN.PACKAGE.BROOM.MIXED

[72] Wickham H François R Henry L Müller K Vaughan D. Dplyr: A Grammar of Data Manipulation [R package version 1.2.1]. CRAN: Contributed Packages. (2026). 10.32614/CRAN.PACKAGE.DPLYR

[73] Wickham H Chang W Henry L Pedersen TL Takahashi K Wilke C Woo K Yutani H Dunnington D van den Brand T. Ggplot2: Create Elegant Data Visualisations Using the Grammar of Graphics [R package version 4.0.2]. CRAN: Contributed Packages. (2026). 10.32614/CRAN.PACKAGE.GGPLOT2

[74] Bates D Maechler M Bolker B Walker S. Lme4: Linear Mixed-Effects Models using “Eigen” and S4 [R package version 1.1-38]. CRAN: Contributed Packages. (2025). 10.32614/CRAN.PACKAGE.LME4

[75] Schauberger P Walker A. Openxlsx: Read, Write and Edit xlsx Files [R package version 4.2.8.1]. CRAN: Contributed Packages. (2025). 10.32614/CRAN.PACKAGE.OPENXLSX

[76] Wickham H Henry L. Purrr: Functional Programming Tools [R package version 1.2.1]. CRAN: Contributed Packages. (2026). 10.32614/CRAN.PACKAGE.PURRR

[77] Wickham H Hester J Bryan J. Readr: Read Rectangular Text Data [R package version 2.1.6]. CRAN: Contributed Packages. (2025). 10.32614/CRAN.PACKAGE.READR

[78] Wickham H Vaughan D Girlich M. Tidyr: Tidy Messy Data [R package version 1.3.2]. CRAN: Contributed Packages. (2025). 10.32614/CRAN.PACKAGE.TIDYR

[79] LaFrance C Garcia LJ Labreche J. The effect of a therapy dog on the communication skills of an adult with aphasia. J Commun Disord. (2007) 40:215–24. 10.1016/J.JCOMDIS.2006.06.010 16950329

[80] Andrade C. The ceiling effect, the floor effect, and the importance of active and placebo control arms in randomized controlled trials of an investigational drug. Indian J Psychol Med. (2021) 43:360. 10.1177/02537176211021280 34385732 PMC8327873

[81] Königs M Beurskens EA Snoep L Scherder EJ Oosterlaan J. Effects of timing and intensity of neurorehabilitation on functional outcome after traumatic brain injury: a systematic review and meta-analysis. Arch Phys Med Rehabil. (2018) 99:1149–1159.e1. 10.1016/J.APMR.2018.01.013 29428344

[82] Hayashi S Kamo T Momosaki R. Effectiveness of early rehabilitation interventions in patients with traumatic brain injury using a large database. PM R. (2025) 17:170–177. 10.1002/PMRJ.13243 39105522

[83] Richter S Stevenson S Newman T Wilson L Maas AIR Nieboer D. Study design features associated with patient attrition in studies of traumatic brain injury: a systematic review. J Neurotrauma. (2020) 37:1845–53. 10.1089/NEU.2020.7000 32345119

[84] Allen VC Windsor TD. Age differences in the use of emotion regulation strategies derived from the process model of emotion regulation: a systematic review. Aging Ment Health. (2019) 23:1–14. 10.1080/13607863.2017.1396575 29148830

[85] Paice L Aleligay A Checklin M. A systematic review of interventions for adults with social communication impairments due to an acquired brain injury: significant other reports. Int J Speech Lang Pathol. (2020) 22:537–48. 10.1080/17549507.2019.1701082 32135070

[86] Finch E Copley A Cornwell P Kelly C. Systematic review of behavioral interventions targeting social communication difficulties after traumatic brain injury. Arch Phys Med Rehabil. (2016) 97:1352–65. 10.1016/J.APMR.2015.11.005 26679234

[87] McGlashan HL Thompson K Lam M Cruwys T Walter ZC Beadle E. Group psychosocial interventions following acquired brain injury: a systematic review and meta-analysis of group process and outcomes. Neuropsychol Rev. (2025):1–36. 10.1007/S11065-025-09670-W 40835805 PMC13653871

[88] Long E Patterson S Maxwell K Blake C Pérez RB Lewis R. COVID-19 pandemic and its impact on social relationships and health. J Epidemiol Community Health. (2022) 76:128–32. 10.1136/JECH-2021-216690 34413184 PMC8380476

[89] Reddy A Resnik L Freburger J Ciolek DE Gifford DR Whitten MJ. Rapid changes in the provision of rehabilitation care in post-acute and long-term care settings during the COVID-19 pandemic. J Am Med Dir Assoc. (2021) 22:2240–4. 10.1016/J.JAMDA.2021.08.022 34534491 PMC8390362

[90] Camicia ME Cournan MC Rye J. COVID-19 and inpatient rehabilitation nursing care: lessons learned and implications for the future. Rehabil Nurs. (2021) 46:187–96. 10.1097/RNJ.0000000000000337 34009902 PMC8270222

[91] Bayly J Bradshaw A Fettes L Omarjee M Talbot-Rice H Walshe C. Understanding the impact of the COVID-19 pandemic on delivery of rehabilitation in specialist palliative care services: an analysis of the CovPall-rehab survey data. Palliat Med. (2022) 36:319–31. 10.1177/02692163211063397 34964384 PMC13033368

[92] Sutter-Leve R Passint E Ness D Rindflesch A. The caregiver experience after stroke in a COVID-19 environment: a qualitative study in inpatient rehabilitation. J Neurol Phys Ther. (2021) 45:14–20. 10.1097/NPT.0000000000000336 33086240 PMC7737698

[93] Citarelli G Garofalo C Esposito MG Torre V Magliano L Politano L. Impact of the COVID-19 pandemic on rehabilitation setting. Part 1: professionals’ views on the changes in routine care provided by a rehabilitation centre for patients with muscle diseases. Acta Myol. (2021) 40:132–4. 10.36185/2532-1900-054 34632295 PMC8489169

[94] Luker S Laver K Lane R Potter E Harrod AM Bierer P. ‘Put in a room and left’: a qualitative study exploring the lived experiences of COVID-19 isolation and quarantine among rehabilitation inpatients. Ann Med. (2023) 55:198–206. 10.1080/07853890.2022.2155698 36538037 PMC9788724

[95] Gorenko JA Moran C Flynn M Dobson K Konnert C. Social isolation and psychological distress among older adults related to COVID-19: a narrative review of remotely-delivered interventions and recommendations. J Appl Gerontol. (2021) 40:3–13. 10.1177/0733464820958550 32914668

[96] Pedrosa AL Bitencourt L Fróes ACF Cazumbá MLB Campos RGB de Brito SBCS. Emotional, behavioral, and psychological impact of the COVID-19 pandemic. Front Psychol. (2020) 11:566212. 10.3389/FPSYG.2020.566212 33117234 PMC7561666

[97] Seckman C. The impact of COVID-19 on the psychosocial well-being of older adults: a literature review. J Nurs Scholarsh. (2023) 55:97–111. 10.1111/JNU.12824 36218196 PMC9874600

[98] Geng EH Powell BJ Goss CW Lewis CC Sales AE Kim B. When the parts are greater than the whole: how understanding mechanisms can advance implementation research. Implement Sci. (2025) 20:22. 10.1186/S13012-025-01427-6 40361178 PMC12070568

[99] Machalicek W Gross DP Armijo-Olivo S Ferriero G Kiekens C Martin R. The role of single case experimental designs in evidence creation in rehabilitation. Eur J Phys Rehabil Med. (2024) 60:1100–11. 10.23736/S1973-9087.24.08713-6 39374052 PMC11729724

[100] Aldawsari KA. Disclosure in the era of generative artificial intelligence. Front Digit Health. (2026) 8:1826808. 10.3389/FDGTH.2026.1826808 42110916 PMC13149261

