# Supplementary material for: Transfer effects of animal-assisted therapy on socioemotional skills in adult inpatient rehabilitation after acquired brain injury: a randomized controlled trial
Source: Front Rehabil Sci. 2026 Sep 3;7:1818862. doi: 10.3389/fresc.2026.1818862 (PMC13584825; doi:10.3389/fresc.2026.1818862)
Supplement: Supplementary file 1 [file Supplementaryfile1.docx]

Supplementary Material

Supplementary Table 1

*Coding Scheme for the Ethogram.*

| **Category** | **Key/Stopp** | **Code** | **Description** |
| --- | --- | --- | --- |
| **Verbal behavior patient** | | | |
| No verbal | 1 | Non-targeted speech acts | - Unfocused verbal expression that does not appear to be directed at a specific recipient - Self-referential statements - Muttering to oneself - Addressee not clearly identifiable |
| Active | 2 | Self-initiated communication with RS | - Speaking without being prompted by the research staff |
| Reactive | 3 | Externally initiated communication with RS | - Responding to a question, statement, or prompt - Speaking or mumbling - Utterances such as “mhm”, “aha” - Consider the context: if the response relates to the topic or question, then code as reactive; otherwise, code as active |
| Active question | 4 | Question directed at RS | - Patient directs a question at the research staff |
| **Non-verbal behavior patient** | | | |
| Body-movement | d/d |  | - Turn/lift the body or face towards RS; lean/move the upper body toward the stimulus - Sit down at the beginning of the conversation - Move the head back and forth between RS - Gesture while speaking - Only code the moment of movement; start: turning towards RS; stop: end of turning, maintaining the position - Movement towards RS is usually followed by eye contact - Note: if the participant has significant movement limitations, attempt to code any attempted movements (as best as possible) - Do not code: pouring water, tidying up or picking up planks |
| Eye-contact | n/n |  | - Eye contact - Focused gaze - Following gaze - When the gaze is directed at RS |
| **Emotions patient** | | | |
| Astonishment | j | Surprise | - Patients are surprised (eyes widen, inhaling breath) - Patients are irritated (pause actions) - Patients are shocked (twitching body) |
| Sadness | b | Sadness | - Upward tilt of the inner eyebrows - Frowning - Downturned corners of the mouth - Verbal expression of sadness |
| Anger | c | Anger, rage | - Lowered eyebrows - Tense lower eyelids - Pressed lips - Shouting or calling out in anger - Swearing, scolding - Direct verbal expression of anger |
| Fear | e | Fear | - Raised upper eyelids, tense lower eyelids - Slightly lifted eyebrows - Lips stretched horizontally - Verbal expression of fear |
| Face not visible | f | Face not visible | - Mouth and/or eyes not visible - Emotion not identifiable due to obstruction - If frequent, comment in the documents |
| Neutral | g | Neutral facial expression | - Face visible, no discernible emotion |
| Positive Emotion | p | Joy, pleasure | - Smiling - Corners of the mouth pulled upward - Cheeks raised - Eyes narrowed - Laughter sounds - Do NOT code if smiling is constant and not accompanied by emotional expression |
| **Social behavior patient** | | | |
| Prosocial Behavior | s/s | Prosocial behavior directed toward one or both RS | - Pouring water for RS - Handing wooden KAPLA® planks to RS - Picking up KAPLA® planks from the floor - Dismantling the tower does not count - Code for the entire duration of the behavior |
| **Events** | | | |
| KAPLA® planks fall | k/k | Individual planks fall (unintended) | - Planks fall off the tower - Planks fall to the floor - The exact stop time is not particularly relevant |
| Water offer | w/w | Request from RS for water | - Research staff asks for water - The exact stopping point is not particularly relevant |
| Tower crashes | t/t | Tower collapses (intended by RS) | - Part of the tower or the entire tower collapses, triggered by one of the research staff - Even if the tower does not collapse completely, code as a crash (as long as no subsequent event occurs in which the tower is completely knocked over) - Stop timing is not highly relevant |
| **Verbal behavior research staff //** Observe both RS simultaneously | | | |
| No verbal RS | v/v |  | See above |
| Active RS | i/i |  | See above |
| Reactive RS | x/x |  | See above |
| Active question RS | y/y |  | See above |
| **Non-verbal behavior research staff** | | | |
| Body movement RS | m/m |  | See above |
| Eye-contact RS | u/u |  | See above |
| **Emotions research staff** | | | |
| Positive emotion RS | o/o |  | See above |
| **Observation on/off** | | | |
| **On** | h |  | - Start: face patient visible |
| **Off** | r |  | - Stopp: RS turns of camera - Planks are cleaned up |

*Note.* Single letters indicate the keyboard key pressed to code the respective variable. Wooden construction planks (KAPLA® planks) were used during the interaction task.
RS = research staff.

Supplementary Table 2

*Baseline (t_0_) Differences Between Study Completers and Non-Completers (*N *= 70).*

| **Outcomes** | **Level** | **Term** | ***n*** | ***difference*** | **95% CI** | | ***p*-value** |
| --- | --- | --- | --- | --- | --- | --- | --- |
|  |  |  |  |  | *LL* | *UL* |  |
| **Behavior analyses** | Social behavior | unadjusted | 47 | 0.64 | −1.76 | 3.05 | .593 |
|  |  | adjusted | 47 | 0.60 | −1.82 | 3.03 | .618 |
|  | Positive emotions | unadjusted | 47 | −5.63 | −20.37 | 9.10 | .445 |
|  |  | adjusted | 47 | −6.00 | −20.71 | 8.71 | .415 |
|  | Negative emotions | unadjusted | 47 | 0.08 | −0.08 | 0.25 | .326 |
|  |  | adjusted | 47 | 0.08 | −0.08 | 0.25 | .309 |
| **BDI−FS** | | unadjusted | 56 | −0.51 | −2.33 | 1.32 | .581 |
|  |  | adjusted | 56 | −0.52 | −2.40 | 1.36 | .584 |
| **MASC** | | unadjusted | 53 | 3.43 | −1.44 | 8.31 | .163 |
|  |  | adjusted | 53 | 3.04 | −1.95 | 8.04 | .227 |
| **FPTM** | Psychological distress | unadjusted | 49 | −0.07 | −0.55 | 0.40 | .764 |
|  |  | adjusted | 49 | −0.07 | −0.55 | 0.41 | .780 |
|  | Hope | unadjusted | 50 | 0.07 | −0.35 | 0.49 | .728 |
|  |  | adjusted | 50 | 0.08 | −0.34 | 0.51 | .694 |
|  | Denial | unadjusted | 50 | −0.48 | −1.01 | 0.06 | .081 |
|  |  | adjusted | 50 | −0.47 | −1.01 | 0.08 | .091 |
|  | Knowledge | unadjusted | 50 | −0.24 | −0.62 | 0.14 | .211 |
|  |  | adjusted | 50 | −0.25 | −0.63 | 0.13 | .190 |
|  | Initiative | unadjusted | 50 | −0.30 | −0.99 | 0.38 | .379 |
|  |  | adjusted | 50 | −0.32 | −1.02 | 0.37 | .355 |
|  | Symptom-related care | unadjusted | 49 | 0.03 | −0.45 | 0.52 | .887 |
|  |  | adjusted | 49 | 0.06 | −0.42 | 0.55 | .797 |
| **WHOQOL-BREF** | Physical Health | unadjusted | 54 | −2.08 | −9.35 | 5.19 | .568 |
|  |  | adjusted | 54 | −1.83 | −9.24 | 5.57 | .621 |
|  | Psychological well-being | unadjusted | 54 | −0.53 | −8.17 | 7.11 | .890 |
|  |  | adjusted | 54 | −0.60 | −8.40 | 7.21 | .879 |
|  | Social relationships | unadjusted | 54 | 0.00 | −10.34 | 10.34 | 1.000 |
|  |  | adjusted | 54 | −0.67 | −11.14 | 9.80 | .898 |
|  | Environment | unadjusted | 52 | 0.62 | −8.35 | 9.60 | .890 |
|  |  | adjusted | 52 | 0.51 | −8.67 | 9.70 | .911 |
| **Therapy adherence** | | unadjusted | 50 | 0.68 | 0.45 | 0.91 | <.001* |
|  |  | adjusted | 50 | 0.69 | 0.46 | 0.92 | <.001* |

*Note.* BDI-FS = Beck Depression Inventory Fast Screen; CI = confidence interval; FPTM = psychotherapy motivation questionnaire (German: Fragebogen zur Psychotherapiemotivation); LL = lower limit of the 95% confidence interval; MASC = Movie for the Assessment of Social Cognition; UL = upper limit of the 95% confidence interval; WHOQOL-BREF = World Health Organization Quality of Life-BREF.

* Indicates statistically significant associations (*p* < .05).

Supplementary Table 3

*Linear Regression Models of Primary and Secondary Outcomes at Post-Assessment (t_1_), Including Covariates (*N *= 44).*

| **Outcomes** | **Level** | **Term** | ***difference*** | **95% *CI*** | | ***p*-value** |
| --- | --- | --- | --- | --- | --- | --- |
|  |  |  |  | *LL* | *UL* |  |
| **Behavior analyses** | Social behavior | Condition | 3.77 | 0.95 | 6.60 | .011* |
|  |  | Baseline | 0.83 | 0.33 | 1.33 | .002* |
|  |  | Gender | −1.28 | −4.29 | 1.74 | .394 |
|  |  | Age | 1.94 | −0.80 | 4.69 | .159 |
|  |  | FIM | −0.03 | −0.10 | 0.05 | .470 |
|  |  | No. of sessions attended | 2.64 | −1.64 | 6.92 | .219 |
|  |  | Social behavior RS | 0.12 | −0.24 | 0.48 | .495 |
|  |  | Positive emotions RS | 0.00 | −0.12 | 0.12 | .940 |
|  | Positive emotions | Condition | −0.24 | −7.96 | 7.48 | .950 |
|  |  | Baseline | 0.07 | −0.14 | 0.27 | .517 |
|  |  | Gender | 1.57 | −6.10 | 9.25 | .679 |
|  |  | Age | 7.41 | 0.36 | 14.45 | .040 |
|  |  | FIM | −0.04 | −0.23 | 0.15 | .672 |
|  |  | No. of sessions attended | 0.77 | −10.22 | 11.76 | .887 |
|  |  | Social behavior RS | 0.14 | −0.70 | 0.97 | .742 |
|  |  | Positive emotions RS | 0.40 | 0.10 | 0.70 | .011* |
|  | Negative emotion | Condition | 0.32 | −0.06 | 0.69 | .093 |
|  |  | Baseline | 0.02 | −0.82 | 0.86 | .965 |
|  |  | Gender | 0.08 | −0.32 | 0.48 | .678 |
|  |  | Age | 0.32 | −0.04 | 0.68 | .080 |
|  |  | FIM | 0.00 | −0.01 | 0.01 | .427 |
|  |  | No. of sessions attended | −0.17 | −0.73 | 0.40 | .554 |
|  |  | Social behavior RS | 0.01 | −0.03 | 0.06 | .526 |
|  |  | Positive emotions RS | −0.01 | −0.02 | 0.01 | .350 |
| **BDI-FS** | | Condition | 0.13 | −1.91 | 2.17 | .898 |
|  |  | Baseline | 0.24 | −0.09 | 0.57 | .147 |
|  |  | Gender | −1.50 | −3.47 | 0.46 | .129 |
|  |  | Age | −0.65 | −2.57 | 1.27 | .494 |
|  |  | FIM | 0.01 | −0.05 | 0.06 | .806 |
|  |  | No. of sessions attended | 0.04 | −3.67 | 3.74 | .983 |
| **MASC** | | Condition | 0.31 | −3.66 | 4.28 | .874 |
|  |  | Baseline | 0.85 | 0.61 | 1.09 | <.001* |
|  |  | Gender | 0.26 | −3.38 | 3.90 | .885 |
|  |  | Age | 2.70 | −1.37 | 6.78 | .185 |
|  |  | FIM | 0.01 | −0.10 | 0.11 | .919 |
|  |  | No. of sessions attended | −2.98 | −13.05 | 7.09 | .549 |
| **FPTM** | Psychological distress | Condition | 0.06 | −0.45 | 0.56 | .816 |
|  |  | Baseline | 0.67 | 0.36 | 0.98 | <.001* |
|  |  | Gender | 0.10 | −0.40 | 0.59 | .696 |
|  |  | Age | −0.31 | −0.80 | 0.19 | .212 |
|  |  | FIM | 0.00 | −0.01 | 0.01 | .994 |
|  |  | No. of sessions attended | 0.19 | −0.70 | 1.08 | .668 |
|  | Hope | Condition | 0.32 | −0.11 | 0.74 | .139 |
|  |  | Baseline | 0.55 | 0.27 | 0.84 | <.001* |
|  |  | Gender | 0.00 | −0.38 | 0.38 | .996 |
|  |  | Age | −0.16 | −0.57 | 0.26 | .444 |
|  |  | FIM | 0.00 | −0.01 | 0.01 | .653 |
|  |  | No. of sessions attended | −0.66 | −1.39 | 0.08 | .080 |
|  | Denial | Condition | −0.18 | −0.73 | 0.37 | .511 |
|  |  | Baseline | 0.64 | 0.29 | 0.99 | .001* |
|  |  | Gender | 0.22 | −0.26 | 0.70 | .353 |
|  |  | Age | 0.03 | −0.52 | 0.58 | .909 |
|  |  | FIM | 0.00 | −0.01 | 0.02 | .503 |
|  |  | No. of sessions attended | −0.05 | −1.01 | 0.91 | .914 |
|  | Knowledge | Condition | −0.14 | −0.81 | 0.53 | .670 |
|  |  | Baseline | 0.40 | −0.07 | 0.87 | .092 |
|  |  | Gender | −0.02 | −0.61 | 0.57 | .945 |
|  |  | Age | −0.54 | −1.18 | 0.10 | .095 |
|  |  | FIM | −0.01 | −0.03 | 0.01 | .256 |
|  |  | No. of sessions attended | −0.66 | −1.81 | 0.49 | .250 |
|  | Initiative | Condition | −0.01 | −0.79 | 0.77 | .974 |
|  |  | Baseline | 0.57 | 0.28 | 0.87 | .001* |
|  |  | Gender | −0.41 | −1.10 | 0.27 | .223 |
|  |  | Age | −0.26 | −1.02 | 0.49 | .485 |
|  |  | FIM | 0.00 | −0.02 | 0.02 | .938 |
|  |  | No. of sessions attended | −0.25 | −1.61 | 1.11 | .713 |
|  | Symptom-related care | Condition | 0.16 | −0.31 | 0.63 | .489 |
|  |  | Baseline | 0.34 | 0.10 | 0.58 | .008* |
|  |  | Gender | −0.24 | −0.66 | 0.18 | .254 |
|  |  | Age | 0.28 | −0.17 | 0.73 | .213 |
|  |  | FIM | 0.01 | 0.00 | 0.02 | .062 |
|  |  | No. of sessions attended | −0.65 | −1.45 | 0.14 | .104 |
| **WHOQOL-BREF** | Physical health | Condition | 0.03 | −6.39 | 6.44 | .994 |
|  |  | Baseline | 0.41 | 0.15 | 0.66 | .003* |
|  |  | Gender | 0.90 | −5.06 | 6.85 | .760 |
|  |  | Age | 2.48 | −4.03 | 8.98 | .443 |
|  |  | FIM | −0.04 | −0.21 | 0.14 | .679 |
|  |  | No. of sessions attended | −8.15 | −20.00 | 3.71 | .171 |
|  | Psychological well-being | Condition | 4.26 | −1.92 | 10.44 | .169 |
|  |  | Baseline | 0.46 | 0.27 | 0.64 | <.001* |
|  |  | Gender | −5.27 | −10.81 | 0.27 | .061 |
|  |  | Age | 5.16 | −0.89 | 11.22 | .092 |
|  |  | FIM | 0.07 | −0.10 | 0.24 | .389 |
|  |  | No. of sessions attended | −0.39 | −11.84 | 11.07 | .945 |
|  | Social relationships | Condition | −7.01 | −18.24 | 4.22 | .212 |
|  |  | Baseline | 0.74 | 0.44 | 1.03 | <.001* |
|  |  | Gender | −0.64 | −10.38 | 9.10 | .894 |
|  |  | Age | −2.32 | −13.08 | 8.43 | .662 |
|  |  | FIM | −0.29 | −0.60 | 0.02 | .069 |
|  |  | No. of sessions attended | 7.72 | −11.73 | 27.16 | .424 |
|  | Environment | Condition | −0.70 | −9.39 | 8.00 | .871 |
|  |  | Baseline | 0.50 | 0.26 | 0.75 | <.001* |
|  |  | Gender | −3.15 | −10.75 | 4.45 | .403 |
|  |  | Age | 6.25 | −2.43 | 14.93 | .151 |
|  |  | FIM | 0.13 | −0.12 | 0.37 | .300 |
|  |  | No. of sessions attended | −11.66 | −26.84 | 3.53 | .127 |
| **Therapy adherence** | | Condition | −1.17 | −3.39 | 1.06 | .297 |
|  |  | Gender | −0.26 | −2.54 | 2.03 | .822 |
|  |  | Age | −0.43 | −2.71 | 1.86 | .707 |
|  |  | FIM | 0.01 | −0.05 | 0.07 | .778 |

*Note.* BDI-FS = Beck Depression Inventory Fast Screen; CI = confidence interval; FIM = Functional Independence Measure; FPTM = psychotherapy motivation questionnaire (German: Fragebogen zur Psychotherapiemotivation); LL = lower limit of the 95% confidence interval; MASC = Movie for the Assessment of Social Cognition; RS = research staff; UL = upper limit of the 95% confidence interval; WHOQOL-BREF = World Health Organization Quality of Life-BREF.

* Indicates statistically significant associations (*p* < .05).

Supplementary Table 4

*Linear Mixed-Effects Models Across Time for Primary and Secondary Outcomes (*N *=44).*

| **Outcomes** | **Level** | **Term** | ***difference*** | **95% *CI*** | |
| --- | --- | --- | --- | --- | --- |
|  |  |  |  | *LL* | *UL* |
| **Behavior analyses** | Social behavior | Condition t_0_ | −0.14 | −1.81 | 1.52 |
|  |  | t_1_ | −1.49 | −3.42 | 0.44 |
|  |  | t_2_ | −1.33 | −3.45 | 0.81 |
|  |  | t_3_ | −0.93 | −3.29 | 1.42 |
|  |  | Baseline | 0.78 | 0.59 | 0.98 |
|  |  | Condition*t_1_ | 3.34 | 1.10 | 5.58 |
|  |  | Condition*t_2_ | 2.33 | −0.13 | 4.77 |
|  |  | Condition*t_3_ | 1.10 | −1.61 | 3.80 |
|  | Positive emotions | Condition t_0_ | −2.82 | −9.98 | 4.34 |
|  |  | t_1_ | −4.50 | −13.02 | 4.06 |
|  |  | t_2_ | −6.09 | −15.46 | 3.34 |
|  |  | t_3_ | −0.66 | −10.95 | 9.77 |
|  |  | Baseline | 0.51 | 0.37 | 0.65 |
|  |  | Condition*t_1_ | 4.31 | −5.64 | 14.21 |
|  |  | Condition*t_2_ | 8.21 | −2.67 | 18.98 |
|  |  | Condition*t_3_ | 5.55 | −6.40 | 17.40 |
|  | Negative emotions | Condition t_0_ | 0.04 | −0.13 | 0.20 |
|  |  | t_1_ | −0.03 | −0.23 | 0.18 |
|  |  | t_2_ | −0.01 | −0.23 | 0.22 |
|  |  | t_3_ | −0.05 | −0.29 | 0.20 |
|  |  | Baseline | 0.24 | 0.02 | 0.46 |
|  |  | Condition*t_1_ | 0.11 | −0.12 | 0.35 |
|  |  | Condition*t_2_ | −0.04 | −0.30 | 0.21 |
|  |  | Condition*t_3_ | 0.02 | −0.26 | 0.30 |
| **BDI-FS** | | Condition t_0_ | −0.02 | −1.04 | 1.01 |
|  |  | t_1_ | −0.86 | −2.16 | 0.43 |
|  |  | t_2_ | 0.13 | −1.39 | 1.66 |
|  |  | t_3_ | −1.38 | −3.00 | 0.23 |
|  |  | Baseline | 0.59 | 0.47 | 0.71 |
|  |  | Condition*t_1_ | 0.35 | −1.18 | 1.89 |
|  |  | Condition*t_2_ | −0.86 | −2.61 | 0.89 |
|  |  | Condition*t_3_ | −0.10 | −1.97 | 1.76 |
| **MASC** | | Condition t_0_ | 0.25 | −1.86 | 2.36 |
|  |  | t_1_ | 3.22 | 0.78 | 5.67 |
|  |  | t_2_ | 2.51 | −0.29 | 5.31 |
|  |  | t_3_ | 3.31 | 0.33 | 6.30 |
|  |  | Baseline | 0.89 | 0.79 | 1.00 |
|  |  | Condition*t_1_ | −0.42 | −3.33 | 2.47 |
|  |  | Condition*t_2_ | −0.15 | −3.41 | 3.11 |
|  |  | Condition*t_3_ | 0.72 | −2.80 | 4.23 |
| **FPTM** | Psychological distress | Condition t_0_ | −0.01 | −0.26 | 0.24 |
|  |  | t_1_ | −0.39 | −0.68 | −0.10 |
|  |  | t_2_ | −0.20 | −0.53 | 0.13 |
|  |  | t_3_ | 0.12 | −0.23 | 0.48 |
|  |  | Baseline | 0.82 | 0.69 | 0.94 |
|  |  | Condition*t_1_ | 0.27 | −0.07 | 0.61 |
|  |  | Condition*t_2_ | 0.09 | −0.29 | 0.47 |
|  |  | Condition*t_3_ | −0.19 | −0.61 | 0.22 |
|  | Hope | Condition t_0_ | −0.04 | −0.27 | 0.19 |
|  |  | t_1_ | −0.12 | −0.41 | 0.18 |
|  |  | t_2_ | −0.03 | −0.37 | 0.30 |
|  |  | t_3_ | −0.26 | −0.61 | 0.09 |
|  |  | Baseline | 0.68 | 0.56 | 0.80 |
|  |  | Condition*t_1_ | 0.35 | 0.00 | 0.69 |
|  |  | Condition*t_2_ | 0.15 | −0.23 | 0.53 |
|  |  | Condition*t_3_ | 0.36 | −0.05 | 0.77 |
|  | Denial | Condition t_0_ | −0.03 | −0.32 | 0.26 |
|  |  | t_1_ | −0.01 | −0.33 | 0.30 |
|  |  | t_2_ | −0.03 | −0.39 | 0.33 |
|  |  | t_3_ | −0.07 | −0.46 | 0.31 |
|  |  | Baseline | 0.80 | 0.66 | 0.94 |
|  |  | Condition*t_1_ | −0.20 | −0.57 | 0.17 |
|  |  | Condition*t_2_ | −0.24 | −0.65 | 0.18 |
|  |  | Condition*t_3_ | −0.19 | −0.63 | 0.26 |
|  | Knowledge | Condition t_0_ | 0.04 | −0.29 | 0.37 |
|  |  | t_1_ | 0.17 | −0.23 | 0.56 |
|  |  | t_2_ | 0.36 | −0.08 | 0.80 |
|  |  | t_3_ | 0.46 | −0.02 | 0.93 |
|  |  | Baseline | 0.69 | 0.49 | 0.90 |
|  |  | Condition*t_1_ | −0.06 | −0.52 | 0.41 |
|  |  | Condition*t_2_ | −0.34 | −0.85 | 0.17 |
|  |  | Condition*t_3_ | −0.23 | −0.78 | 0.32 |
|  | Initiative | Condition t_0_ | 0.04 | −0.35 | 0.43 |
|  |  | t_1_ | −0.27 | −0.73 | 0.18 |
|  |  | t_2_ | −0.30 | −0.82 | 0.22 |
|  |  | t_3_ | −0.17 | −0.72 | 0.38 |
|  |  | Baseline | 0.78 | 0.65 | 0.92 |
|  |  | Condition*t_1_ | 0.08 | −0.45 | 0.62 |
|  |  | Condition*t_2_ | 0.09 | −0.51 | 0.68 |
|  |  | Condition*t_3_ | −0.01 | −0.65 | 0.63 |
|  | Symptom-related care | Condition t_0_ | −0.09 | −0.36 | 0.17 |
|  |  | t_1_ | −0.02 | −0.34 | 0.31 |
|  |  | t_2_ | −0.18 | −0.53 | 0.17 |
|  |  | t_3_ | −0.09 | −0.46 | 0.28 |
|  |  | Baseline | 0.65 | 0.53 | 0.78 |
|  |  | Condition*t_1_ | 0.22 | −0.16 | 0.59 |
|  |  | Condition*t_2_ | 0.39 | −0.02 | 0.79 |
|  |  | Condition*t_3_ | 0.34 | −0.09 | 0.78 |
| **WHOQOL-BREF** | Physical health | Condition t_0_ | −0.57 | −4.34 | 3.20 |
|  |  | t_1_ | 0.93 | −3.84 | 5.70 |
|  |  | t_2_ | −3.49 | −9.10 | 2.08 |
|  |  | t_3_ | −6.93 | −12.84 | −1.01 |
|  |  | Baseline | 0.69 | 0.59 | 0.80 |
|  |  | Condition*t_1_ | 0.05 | −5.65 | 5.74 |
|  |  | Condition*t_2_ | 3.08 | −3.34 | 9.52 |
|  |  | Condition*t_3_ | 7.22 | 0.33 | 14.14 |
|  | Psychological well-being | Condition t_0_ | 0.15 | −3.96 | 4.26 |
|  |  | t_1_ | 1.70 | −3.58 | 7.00 |
|  |  | t_2_ | −1.28 | −7.50 | 4.88 |
|  |  | t_3_ | 0.19 | −6.37 | 6.72 |
|  |  | Baseline | 0.57 | 0.47 | 0.67 |
|  |  | Condition*t_1_ | 1.02 | −5.31 | 7.33 |
|  |  | Condition*t_2_ | 5.60 | −1.49 | 12.77 |
|  |  | Condition*t_3_ | 4.65 | −2.96 | 12.32 |
|  | Social relationships | Condition t_0_ | 1.32 | −5.08 | 7.71 |
|  |  | t_1_ | 6.80 | −0.93 | 14.52 |
|  |  | t_2_ | 13.36 | 4.23 | 22.45 |
|  |  | t_3_ | 11.75 | 2.09 | 21.39 |
|  |  | Baseline | 0.69 | 0.56 | 0.83 |
|  |  | Condition*t_1_ | −7.47 | −16.68 | 1.77 |
|  |  | Condition*t_2_ | −12.46 | −22.90 | −1.96 |
|  |  | Condition*t_3_ | −9.01 | −20.25 | 2.24 |
|  | Environment | Condition t_0_ | 0.19 | −4.25 | 4.64 |
|  |  | t_1_ | 6.37 | 0.68 | 12.06 |
|  |  | t_2_ | 4.60 | −2.31 | 11.41 |
|  |  | t_3_ | 2.63 | −4.71 | 9.95 |
|  |  | Baseline | 0.74 | 0.63 | 0.84 |
|  |  | Condition*t_1_ | −7.14 | −13.85 | −0.40 |
|  |  | Condition*t_2_ | −4.07 | −11.80 | 3.77 |
|  |  | Condition*t_3_ | −2.03 | −10.46 | 6.41 |

*Note.* AAT = animal-assisted therapy; BDI-FS = Beck Depression Inventory Fast Screen; CI = 95% confidence interval; FPTM = psychotherapy motivation questionnaire (German: Fragebogen zur Psychotherapiemotivation); LL = lower limit of the 95% confidence interval; MASC = Movie for the Assessment of Social Cognition; TAU = treatment as usual; t_0_ = baseline; t_1_ = post-assessment; t_2_ = follow-up I; t_3_ = follow-up II; UL = upper limit of the 95% confidence interval; WHOQOL-BREF = World Health Organization Quality of Life-BREF.

Supplementary Table 5

*Linear Mixed-Effects Models Across Time for Primary and Secondary Outcomes, Including Covariates (*N *= 44).*

| **Outcomes** | **Level** | **Term** | ***difference*** | **95% *CI*** | |
| --- | --- | --- | --- | --- | --- |
|  |  |  |  | *LL* | *UL* |
| **Behavior analyses** | Social behavior | Condition t_0_ | 0.32 | −1.31 | 1.95 |
|  |  | t_1_ | −1.57 | −3.44 | 0.27 |
|  |  | t_2_ | −1.00 | −3.04 | 1.05 |
|  |  | t_3_ | −0.56 | −2.89 | 1.65 |
|  |  | Baseline | 0.70 | 0.50 | 0.89 |
|  |  | Gender | −0.02 | −1.24 | 1.19 |
|  |  | Age | 0.81 | −0.29 | 1.90 |
|  |  | FIM | 0.01 | −0.02 | 0.04 |
|  |  | No. of sessions attended | 0.29 | 0.14 | 0.45 |
|  |  | Social behavior RS | 0.03 | −0.01 | 0.07 |
|  |  | Positive emotions RS | 3.24 | 1.10 | 5.40 |
|  |  | Condition*t_1_ | 1.93 | −0.47 | 4.26 |
|  |  | Condition*t_2_ | 0.64 | −1.92 | 3.29 |
|  |  | Condition*t_3_ | 0.32 | −1.31 | 1.95 |
|  | Positive emotions | t_1_ | −2.03 | −9.62 | 5.52 |
|  |  | t_2_ | −4.18 | −13.23 | 4.93 |
|  |  | t_3_ | −6.60 | −16.28 | 3.16 |
|  |  | Baseline | 0.07 | −10.38 | 10.86 |
|  |  | Gender | 0.48 | 0.34 | 0.60 |
|  |  | Age | 1.03 | −4.10 | 6.16 |
|  |  | FIM | 3.28 | −1.14 | 7.64 |
|  |  | No. of sessions attended | −0.02 | −0.13 | 0.09 |
|  |  | Social behavior RS | 0.48 | −5.78 | 6.94 |
|  |  | Positive emotions RS | 0.08 | −0.55 | 0.73 |
|  |  | Condition*t_1_ | 0.25 | 0.08 | 0.44 |
|  |  | Condition*t_2_ | 4.31 | −6.18 | 14.68 |
|  |  | Condition*t_3_ | 9.31 | −1.94 | 20.34 |
|  | Negative emotions | t_1_ | 0.09 | −0.79 | 0.17 |
|  |  | t_2_ | −0.04 | −0.09 | 0.26 |
|  |  | t_3_ | 0.02 | −0.25 | 0.17 |
|  |  | Baseline | −0.04 | −0.21 | 0.24 |
|  |  | Gender | 0.23 | −0.29 | 0.21 |
|  |  | Age | 0.02 | −0.02 | 0.48 |
|  |  | FIM | 0.09 | −0.11 | 0.14 |
|  |  | No. of sessions attended | 0.00 | −0.01 | 0.19 |
|  |  | Social behavior RS | 0.00 | 0.00 | 0.00 |
|  |  | Positive emotions RS | 0.01 | −0.15 | 0.15 |
|  |  | Condition*t_1_ | 0.00 | 0.00 | 0.03 |
|  |  | Condition*t_2_ | 0.11 | −0.01 | 0.00 |
|  |  | Condition*t_3_ | −0.07 | −0.13 | 0.36 |
| **BDI-FS** | | t_1_ | −0.10 | −1.37 | 1.18 |
|  |  | t_2_ | −0.85 | −2.32 | 0.58 |
|  |  | t_3_ | 0.11 | −1.55 | 1.74 |
|  |  | Baseline | −1.41 | −3.16 | 0.31 |
|  |  | Gender | 0.54 | 0.40 | 0.67 |
|  |  | Age | −0.49 | −1.40 | 0.42 |
|  |  | FIM | −0.01 | −0.89 | 0.87 |
|  |  | No. of sessions attended | 0.00 | −0.03 | 0.02 |
|  |  | Condition*t_1_ | 0.16 | −1.06 | 1.40 |
|  |  | Condition*t_2_ | 0.34 | −1.34 | 2.06 |
|  |  | Condition*t_3_ | −0.81 | −2.67 | 1.08 |
| **MASC** | | t_1_ | 0.45 | −2.34 | 3.25 |
|  |  | t_2_ | 3.92 | 1.21 | 6.69 |
|  |  | t_3_ | 2.74 | −0.28 | 5.75 |
|  |  | Baseline | 3.51 | 0.35 | 6.75 |
|  |  | Gender | 0.88 | 0.75 | 1.02 |
|  |  | Age | 0.01 | −2.23 | 2.24 |
|  |  | FIM | 0.27 | −2.02 | 2.57 |
|  |  | No. of sessions attended | 0.00 | −0.05 | 0.06 |
|  |  | Condition*t_1_ | −0.35 | −3.61 | 2.91 |
|  |  | Condition*t_2_ | −1.15 | −4.39 | 2.02 |
|  |  | Condition*t_3_ | −0.39 | −3.85 | 3.10 |
| **FPTM** | Psychological distress | t_1_ | −0.15 | −0.45 | 0.16 |
|  |  | t_2_ | −0.41 | −0.73 | −0.09 |
|  |  | t_3_ | −0.25 | −0.60 | 0.10 |
|  |  | Baseline | 0.06 | −0.30 | 0.44 |
|  |  | Gender | 0.78 | 0.63 | 0.93 |
|  |  | Age | 0.04 | −0.19 | 0.26 |
|  |  | FIM | −0.21 | −0.43 | 0.02 |
|  |  | No. of sessions attended | 0.00 | −0.01 | 0.01 |
|  |  | Condition*t_1_ | 0.16 | −0.15 | 0.47 |
|  |  | Condition*t_2_ | 0.27 | −0.10 | 0.65 |
|  |  | Condition*t_3_ | 0.16 | −0.24 | 0.56 |
|  | Hope | t_1_ | −0.09 | −0.37 | 0.20 |
|  |  | t_2_ | −0.22 | −0.54 | 0.11 |
|  |  | t_3_ | −0.05 | −0.42 | 0.30 |
|  |  | Baseline | −0.28 | −0.67 | 0.09 |
|  |  | Gender | 0.68 | 0.56 | 0.81 |
|  |  | Age | −0.05 | −0.23 | 0.13 |
|  |  | FIM | −0.08 | −0.26 | 0.11 |
|  |  | No. of sessions attended | 0.00 | −0.01 | 0.00 |
|  |  | Condition*t_1_ | −0.03 | −0.30 | 0.25 |
|  |  | Condition*t_2_ | 0.44 | 0.05 | 0.82 |
|  |  | Condition*t_3_ | 0.16 | −0.24 | 0.57 |
|  | Denial | t_1_ | −0.02 | −0.38 | 0.35 |
|  |  | t_2_ | 0.00 | −0.35 | 0.35 |
|  |  | t_3_ | −0.02 | −0.40 | 0.36 |
|  |  | Baseline | −0.06 | −0.46 | 0.34 |
|  |  | Gender | 0.78 | 0.60 | 0.95 |
|  |  | Age | 0.21 | −0.06 | 0.48 |
|  |  | FIM | 0.00 | −0.29 | 0.29 |
|  |  | No. of sessions attended | 0.00 | −0.01 | 0.01 |
|  |  | Condition*t_1_ | −0.10 | −0.48 | 0.28 |
|  |  | Condition*t_2_ | −0.21 | −0.61 | 0.19 |
|  |  | Condition*t_3_ | −0.27 | −0.70 | 0.17 |
|  | Knowledge | t_1_ | −0.03 | −0.43 | 0.38 |
|  |  | t_2_ | 0.18 | −0.26 | 0.61 |
|  |  | t_3_ | 0.37 | −0.11 | 0.84 |
|  |  | Baseline | 0.46 | −0.05 | 0.95 |
|  |  | Gender | 0.69 | 0.47 | 0.90 |
|  |  | Age | 0.12 | −0.16 | 0.40 |
|  |  | FIM | −0.07 | −0.36 | 0.22 |
|  |  | No. of sessions attended | 0.00 | −0.01 | 0.00 |
|  |  | Condition*t_1_ | −0.25 | −0.66 | 0.15 |
|  |  | Condition*t_2_ | 0.00 | −0.49 | 0.52 |
|  |  | Condition*t_3_ | −0.28 | −0.82 | 0.27 |
|  | Initiative | t_1_ | −0.01 | −0.50 | 0.48 |
|  |  | t_2_ | −0.16 | −0.67 | 0.34 |
|  |  | t_3_ | −0.24 | −0.79 | 0.31 |
|  |  | Baseline | −0.11 | −0.71 | 0.46 |
|  |  | Gender | 0.76 | 0.62 | 0.91 |
|  |  | Age | −0.22 | −0.56 | 0.13 |
|  |  | FIM | −0.09 | −0.45 | 0.27 |
|  |  | No. of sessions attended | 0.00 | −0.01 | 0.01 |
|  |  | Condition*t_1_ | −0.20 | −0.70 | 0.28 |
|  |  | Condition*t_2_ | −0.02 | −0.60 | 0.58 |
|  |  | Condition*t_3_ | 0.06 | −0.56 | 0.70 |
|  | Symptom-related care | t_1_ | −0.01 | −0.34 | 0.32 |
|  |  | t_2_ | 0.07 | −0.29 | 0.43 |
|  |  | t_3_ | −0.12 | −0.50 | 0.26 |
|  |  | Baseline | −0.03 | −0.43 | 0.37 |
|  |  | Gender | 0.62 | 0.50 | 0.74 |
|  |  | Age | −0.21 | −0.43 | 0.02 |
|  |  | FIM | 0.16 | −0.06 | 0.38 |
|  |  | No. of sessions attended | 0.00 | 0.00 | 0.01 |
|  |  | Condition*t_1_ | −0.11 | −0.42 | 0.20 |
|  |  | Condition*t_2_ | 0.12 | −0.29 | 0.54 |
|  |  | Condition*t_3_ | 0.34 | −0.09 | 0.77 |
| **WHOQOL-BREF** | Physical health | t_1_ | −0.31 | −4.88 | 4.30 |
|  |  | t_2_ | 0.65 | −4.73 | 6.01 |
|  |  | t_3_ | −3.23 | −9.29 | 2.76 |
|  |  | Baseline | −6.58 | −12.83 | −0.13 |
|  |  | Gender | 0.67 | 0.55 | 0.78 |
|  |  | Age | −1.40 | −4.40 | 1.63 |
|  |  | FIM | 1.24 | −1.74 | 4.26 |
|  |  | No. of sessions attended | −0.03 | −0.11 | 0.05 |
|  |  | Condition*t_1_ | −2.03 | −6.54 | 2.42 |
|  |  | Condition*t_2_ | 0.18 | −6.13 | 6.54 |
|  |  | Condition*t_3_ | 2.66 | −4.21 | 9.61 |
|  | Psychological well-being | t_1_ | 1.37 | −3.73 | 6.51 |
|  |  | t_2_ | 1.79 | −4.32 | 7.89 |
|  |  | t_3_ | −0.93 | −7.92 | 5.78 |
|  |  | Baseline | 0.70 | −6.53 | 7.87 |
|  |  | Gender | 0.56 | 0.46 | 0.67 |
|  |  | Age | −0.72 | −3.94 | 2.51 |
|  |  | FIM | 2.82 | −0.37 | 6.03 |
|  |  | No. of sessions attended | −0.03 | −0.12 | 0.05 |
|  |  | Condition*t_1_ | 0.14 | −4.75 | 5.07 |
|  |  | Condition*t_2_ | 1.21 | −6.01 | 8.39 |
|  |  | Condition*t_3_ | 5.55 | −2.17 | 13.51 |
|  | Social relationships | t_1_ | 1.26 | −6.30 | 8.90 |
|  |  | t_2_ | 5.38 | −2.47 | 13.22 |
|  |  | t_3_ | 13.66 | 4.80 | 22.57 |
|  |  | Baseline | 11.89 | 2.57 | 21.34 |
|  |  | Gender | 0.72 | 0.57 | 0.88 |
|  |  | Age | −2.70 | −8.18 | 2.79 |
|  |  | FIM | −0.70 | −6.22 | 4.86 |
|  |  | No. of sessions attended | −0.10 | −0.25 | 0.05 |
|  |  | Condition*t_1_ | −1.36 | −8.93 | 6.23 |
|  |  | Condition*t_2_ | −6.35 | −15.59 | 2.91 |
|  |  | Condition*t_3_ | −10.97 | −21.18 | −0.82 |
|  | Environment | t_1_ | 1.59 | −3.86 | 7.09 |
|  |  | t_2_ | 5.91 | −0.52 | 12.37 |
|  |  | t_3_ | 5.38 | −2.08 | 12.68 |
|  |  | Baseline | 3.47 | −4.30 | 11.44 |
|  |  | Gender | 0.74 | 0.63 | 0.84 |
|  |  | Age | −2.54 | −6.06 | 0.97 |
|  |  | FIM | 1.73 | −1.85 | 5.32 |
|  |  | No. of sessions attended | 0.07 | −0.02 | 0.17 |
|  |  | Condition*t_1_ | −0.34 | −5.46 | 4.78 |
|  |  | Condition*t_2_ | −6.02 | −13.52 | 1.51 |
|  |  | Condition*t_3_ | −4.28 | −12.50 | 4.17 |

*Note.* BDI-FS = Beck Depression Inventory Fast Screen; CI = confidence interval; FIM = Functional Independence Measure; FPTM = psychotherapy motivation questionnaire (German: Fragebogen zur Psychotherapiemotivation); LL = lower limit of the 95% confidence interval; MASC = Movie for the Assessment of Social Cognition; RS = research staff; UL = upper limit of the 95% confidence interval; WHOQOL-BREF = World Health Organization Quality of Life-BREF.

Supplementary Table 6

*Sensitivity Analyses Examining the Association Between the Number of Animal Species Exposure and Primary and Secondary Outcomes Within the Animal-Assisted Therapy Group.*

| **Outcomes** | **Level** | ***n*** | ***estimate*** | **95% CI** | | ***p*-value** |
| --- | --- | --- | --- | --- | --- | --- |
|  |  |  |  | *LL* | *UL* |  |
| **Behavior analyses** | Social behavior | 30 | 1.30 | −3.33 | 5.93 | .568 |
|  | Positive emotions | 30 | 0.73 | −8.12 | 9.57 | .868 |
|  | Negative emotions | 30 | 0.08 | −0.60 | 0.76 | .809 |
| **BDI-FS** | | 28 | 0.38 | −2.91 | 3.68 | .813 |
| **MASC** | | 25 | −0.80 | −5.92 | 4.32 | .748 |
| **FPTM** | Psychological distress | 25 | 0.61 | 0.11 | 1.10 | .018* |
|  | Hope | 26 | −0.09 | −0.50 | 0.33 | .663 |
|  | Denial | 26 | 0.59 | −0.12 | 1.29 | .100 |
|  | Knowledge | 26 | 0.23 | −0.77 | 1.23 | .640 |
|  | Initiative | 26 | 0.34 | −0.41 | 1.09 | .359 |
|  | Symptom-related care | 26 | 0.23 | −0.51 | 0.97 | .526 |
| **WHOQOL-BREF** | Physical health | 26 | −0.27 | −8.99 | 8.46 | .950 |
|  | Psychological well-being | 26 | −1.31 | −10.31 | 7.70 | .767 |
|  | Social relationships | 26 | 6.39 | −10.12 | 22.89 | .432 |
|  | Environment | 26 | −13.26 | −24.02 | −2.50 | .018* |
| **Adherence to treatment** | | 34 | 0.00 | 0.00 | 0.00 | 1.000 |

*Note.* Estimates represent the difference in post-assessment outcomes for AAT participants exposed to two or more animal species compared with those exposed to one animal species, controlling for baseline levels. Exposure to one animal species was used as the reference category.
AAT = animal-assisted therapy; BDI-FS = Beck Depression Inventory Fast Screen; CI = confidence interval; FPTM = psychotherapy motivation questionnaire (German: Fragebogen zur Psychotherapiemotivation); LL = lower limit of the 95% confidence interval; MASC = Movie for the Assessment of Social Cognition; UL = upper limit of the 95% confidence interval; WHOQOL-BREF = World Health Organization Quality of Life-BREF.

* Indicates statistically significant associations (*p* < .05).

Supplementary Table 7

*Exploratory Analyses of Social Behavior and Negative Emotions at Post-Assessment (t_1_) (N = 44).*

| **Outcomes** | **Term** | ***difference*** | **95% *CI*** | | ***p*-value** |
| --- | --- | --- | --- | --- | --- |
|  |  |  | *LL* | *UL* |  |
| **Social behavior** | Condition | −0.00 | −0.00 | −0.00 | .046* |
|  | Active vocalization | 0.33 | 0.33 | 0.33 | <.001* |
|  | Baseline active vocalization | −0.00 | −0.00 | 0.00 | .148 |
|  | Reactive vocalization | 0.33 | 0.33 | 0.33 | <.001* |
|  | Baseline vocalization | −0.00 | −0.00 | 0.00 | .148 |
|  | Active questions | 0.33 | 0.33 | 0.33 | <.001* |
|  | Baseline active questions | −0.00 | −0.00 | 0.00 | .149 |
|  | Undefined vocalization | 0.33 | 0.33 | 0.33 | <.001* |
|  | Baseline undefined vocalization | −0.00 | −0.00 | 0.00 | .148 |
|  | Eye contact | 0.33 | 0.33 | 0.33 | <.001* |
|  | Baseline eye contact | 0.00 | −0.00 | 0.00 | .563 |
|  | Body movement | 0.33 | 0.33 | 0.33 | <.001* |
|  | Baseline body movement | −0.00 | −0.00 | 0.00 | .617 |
|  | Prosocial behavior | 0.33 | 0.33 | 0.33 | <.001* |
|  | Baseline prosocial behavior | −0.00 | −0.00 | 0.00 | .243 |
| **Negative emotions** | Condition | −0.00 | −0.00 | 0.00 | .389 |
|  | Astonishment | 0.25 | 0.25 | 0.25 | <.001* |
|  | Baseline astonishment | 0.00 | 0.00 | 0.00 | .470 |
|  | Sadness | 0.25 | 0.25 | 0.25 | <.001* |
|  | Baseline sadness | 0.00 | 0.00 | 0.00 | .665 |
|  | Anger | 0.25 | 0.25 | 0.25 | <.001* |
|  | Baseline anger | 0.00 | 0.00 | 0.00 | .788 |
|  | Fear | 0.25 | 0.25 | 0.25 | <.001* |
|  | Baseline fear | 0.00 | 0.00 | 0.00 | .672 |

*Note.* CI = confidence interval; LL = lower limit of the 95% confidence interval; UL = upper limit of the 95% confidence interval.

* Indicates statistically significant associations (*p* < .05).
